# Surveillance of Listeria monocytogenes: Early Detection, Population Dynamics, and Quasimetagenomic Sequencing during Selective Enrichment

**DOI:** 10.1128/AEM.01774-21

**Published:** 2021-11-24

**Authors:** Eva Wagner, Annette Fagerlund, Solveig Langsrud, Trond Møretrø, Merete Rusås Jensen, Birgitte Moen

**Affiliations:** a Nofima —Norwegian Institute of Food, Fisheries and Aquaculture Research, Ås, Norway; The Pennsylvania State University

**Keywords:** *Listeria*, *L. monocytogenes*, quasimetagenomics, Nanopore, Illumina, shotgun sequencing, IMS, immunomagnetic separation, MDA, multiple displacement amplification, selective enrichment, qPCR, population dynamics, ISO 11290-1, qualitative detection, background microbiota

## Abstract

In this study, we addressed different aspects regarding the implementation of quasimetagenomic sequencing as a hybrid surveillance method in combination with enrichment for early detection of Listeria monocytogenes in the food industry. Different experimental enrichment cultures were used, comprising seven L. monocytogenes strains of different sequence types (STs), with and without a background microbiota community. To assess whether the proportions of the different STs changed over time during enrichment, the growth and population dynamics were assessed using *dapE* colony sequencing and *dapE* and 16S rRNA amplicon sequencing. There was a tendency of some STs to have a higher relative abundance during the late stage of enrichment when L. monocytogenes was enriched without background microbiota. When coenriched with background microbiota, the population dynamics of the different STs was more consistent over time. To evaluate the earliest possible time point during enrichment that allows the detection of L. monocytogenes and at the same time the generation of genetic information that enables an estimation regarding the strain diversity in a sample, quasimetagenomic sequencing was performed early during enrichment in the presence of the background microbiota using Oxford Nanopore Technologies Flongle and Illumina MiSeq sequencing. The application of multiple displacement amplification (MDA) enabled detection of L. monocytogenes (and the background microbiota) after only 4 h of enrichment using both applied sequencing approaches. The MiSeq sequencing data additionally enabled the prediction of cooccurring L. monocytogenes strains in the samples.

**IMPORTANCE** We showed that a combination of a short primary enrichment combined with MDA and Nanopore sequencing can accelerate the traditional process of cultivation and identification of L. monocytogenes. The use of Illumina MiSeq sequencing additionally allowed us to predict the presence of cooccurring L. monocytogenes strains. Our results suggest quasimetagenomic sequencing is a valuable and promising hybrid surveillance tool for the food industry that enables faster identification of L. monocytogenes during early enrichment. Routine application of this approach could lead to more efficient and proactive actions in the food industry that prevent contamination and subsequent product recalls and food destruction, economic and reputational losses, and human listeriosis cases.

## INTRODUCTION

As the causative agent of listeriosis, Listeria monocytogenes is one of the most concerning foodborne human bacterial pathogens. Listeriosis is predominantly acquired upon the consumption of contaminated ready-to-eat food products and affects high-risk patient groups such as infants and pregnant, immunocompromised, and elderly people. Symptoms range from a febrile gastroenteritis to a systemic infection resulting in septicemia, meningoencephalitis, placentitis, abortion, or still birth. Contamination of final food products with the pathogen mainly occurs through cross-contamination in the food processing environment (1, 2). The prevalence of L. monocytogenes strains varies between different niches. Some sequence types (STs), such as ST1, ST2, ST4, and ST6, are predominantly associated with human listeriosis cases but only sporadically associated with occurrence in food processing environments and food products. Other STs, such as ST7, ST8, ST9, and ST121, are highly abundant and have been reported to persist in food processing environments over extended periods of time, while they hardly ever cause human listeriosis. The underlying causes behind these differences are not fully understood but have been explained by different degrees of environmental adaptation, stress response, and virulence potential caused by specific genetic traits (3–6).

It is widely acknowledged that L. monocytogenes surveillance in food is not managed by end product monitoring but rather by monitoring of raw materials and the food processing environment to find sources, reservoirs, and cross-contamination routes (7, 8). As L. monocytogenes is abundantly found in the outer environment, sporadic contamination of raw materials or the food processing environment is expected, and a positive sample may be a sporadic event. However, it could also be caused by occasional detachment of persistent clones from a reservoir or originate from a supplier with an ongoing L. monocytogenes problem (9, 10). The first responses to positive samples are often more thorough cleaning of the equipment upstream of the sample site followed by more sampling to identify the source (seek and destroy strategy) (11). Some manufacturers respond by dismantling all of the machines and cleaning and disinfecting the whole production line, even though L. monocytogenes may not originate from the machines; thus, these strategies may not necessarily solve the problem. Sometimes the process of identifying and removing potential reservoirs takes weeks or months. This can be due to complicated product lines, ineffective implemented measures, or ultimately because L. monocytogenes is difficult to eliminate. Meanwhile, production is continued and products are consumed, since positive findings on non-product contact sites in the processing environment do not (and should not) initiate withdrawal of products (12, 13).

The currently employed protocols for the detection of foodborne bacterial pathogens from raw materials, environmental samples, and food products involve the isolation of the respective bacteria from enrichment cultures and subsequent confirmatory biochemical or molecular identification. These methods are labor-intensive and take several days to obtain conclusive results. To recover the foodborne human pathogen L. monocytogenes from a food or environmental sample according to ISO 11290-1 (14), a primary enrichment in Half Fraser broth at 30°C for 24 h and a subsequent secondary enrichment in Full Fraser broth at 37°C for 24 h is required. Both enrichment steps are followed by incubation on selective agar(s) for 48 h each and confirmation of presumptive L. monocytogenes and *Listeria* spp. colonies involving classical microbiological and biochemical identification tests, such as hemolysis, motility, or catalase tests. Today, information about the presumptive presence or absence of L. monocytogenes can be obtained at the earliest 1 to 2 days after the sample has been processed in a laboratory. Obtaining further genetic subtyping information enabling comparison with historical data takes another 2 to 4 days. Such an expenditure of time is barely compatible with modern food production and processing circumstances, as not only presumptive L. monocytogenes but also genetic typing results should optimally be available at latest 12 h after sampling to be able to take action before the next working day. Efforts have been made to shorten the time from sampling to detection by validating alternative protocols against ISO 11290-1, for example, by performing qualitative real-time PCR following primary enrichment after 24 h, thereby omitting secondary enrichment (15). The time from sampling to detection and subsequent subtyping should be as short as possible for L. monocytogenes both in terms of preventing listeriosis outbreaks and fatalities as well as substantial economic losses and food waste. Standard detection methods, however, are designed to confirm the presence or absence of the pathogen but neglect the generation of genetic information, which is required for subtyping and subsequent source tracking. In order to move toward a situation where the management of L. monocytogenes is not based only on the presence or absence of the pathogen in a sample and where genetic typing information is analyzed retrospectively, food manufacturers and quality managers need a methodological pipeline to rapidly distinguish between sporadic contamination, reoccurring contamination from raw materials or other sources, and persistence of certain clones. To enable better pathogen source tracking and quality surveillance, the food industry needs to test and implement new methods and approaches to increase the speed and resolution of pathogen detection and microbiome surveillance (16).

Whole-genome sequencing (WGS) has become a valuable tool for source tracking of L. monocytogenes. Routine sequencing applications in the future promise to reduce both time and expenses for the industry as well as for public health authorities, as several subtyping methods, such as serotyping, multilocus sequence typing (MLST), single-nucleotide polymorphism (SNP) analysis, and resistance and virulence gene profiling, can be performed simultaneously (17, 18). The WGS approach, however, requires the cultivation of an individual isolate prior to sequencing. Thus, the acquisition of timely WGS data in a food industry setting is challenged both by the time required for obtaining the single isolate and the time required for sequencing and sequencing analysis. The appearance of the Oxford Nanopore Technologies (ONT) portable sequencing devices addressed the issue of sequencing time and supported the development of rapid real-time sequencing and analysis pipelines for whole-genome and metagenomic sequencing data, thereby allowing pathogen detection and characterization on-site the moment the data are being generated. Metagenomic shotgun sequencing has previously been used for isolation-independent detection and subtyping of Shiga toxin-producing Escherichia coli (STEC) from spinach (19, 20). The conventional metagenomics approach, however, can be impractical, if not ineffective, for detecting low levels of bacterial pathogen contaminants in food samples and does not discriminate between live and dead cells.

In addition to the aforementioned challenge of time entailed by a culture-dependent approach, another challenge is the potential loss of diversity upon selecting a single colony for WGS, since different L. monocytogenes subtypes may be present in the same sample (21, 22). Additionally, the prevalence of various L. monocytogenes strains of different STs in a sample in the absence or presence of coisolated background microbiota during enrichment depends on multiple factors, one of which might be the enrichment procedure itself (23–25). To address these challenges, quasimetagenomic sequencing, a term that has been applied for the generation of sequencing data from enrichment cultures, could be implemented. This approach has previously been applied for L. monocytogenes, Salmonella enterica, and E. coli in food samples (26–29). Quasimetagenomic sequencing during enrichment promises to increase the resolution and speed of pathogen tracking and could overcome current limitations. However, several obstacles have to be overcome before the approach can be implemented in practical settings. It can be challenging to obtain reasonable results, especially at early time points, due to low pathogen concentrations resulting in low target DNA concentrations. This can be circumvented by additional target enrichment strategies, such as immunomagnetic separation (IMS) for target pathogens, an approach that was previously demonstrated to enrich Salmonella from food samples (28, 30). Another efficient option to increase the amount of target DNA is multiple displacement amplification (MDA), which employs a highly processive bacteriophage DNA polymerase with excellent proofreading activity to perform an isothermal DNA amplification (31–33).

In this study, we addressed different aspects regarding the implementation of quasimetagenomic sequencing as a hybrid surveillance method during enrichment of L. monocytogenes according to ISO 11290-1. The aim was to evaluate the impact of culture enrichment (Half and Full Fraser) on the population dynamics of seven different L. monocytogenes STs frequently isolated from food processing environments in the presence or absence of background microbiota. Furthermore, we examined how this dynamics is represented by colony sequencing compared to targeted amplicon sequencing (*dapE* and 16S rRNA gene). In addition, we wanted to evaluate the use of quantitative PCR (qPCR), IMS, and MDA for low biomass samples and compare low-cost, real-time Nanopore Flongle to Illumina MiSeq sequencing for early detection of L. monocytogenes and detection of cooccurring strains.

## RESULTS

### Population dynamics in L. monocytogenes cultures during enrichment.

The population dynamics of seven L. monocytogenes strains belonging to different STs was assessed throughout the commonly used enrichment protocol for L. monocytogenes and *Listeria* species detection in the food chain, the ISO 11290-1 standard (14), after 4 h, 8 h, and 24 h of primary enrichment in Half Fraser and after 24 h and 48 h of secondary enrichment in Full Fraser. The strains were isolated from three different Norwegian meat processing facilities, comprising both sporadic (ST18, ST19, and ST394) and persistent (ST7, ST8, ST9, and ST121) STs, and were distinguishable by their *dapE* MLST gene sequences (Table 1) (34).

**TABLE 1:** Strains used in the current study

Strain	Species	Source (and ST when available)
Listeria monocytogenes ^*a*^		
MF4536	L. monocytogenes	Meat processing plant (ST9)
MF4565	L. monocytogenes	Meat processing plant (ST18)
MF5376	L. monocytogenes	Meat processing plant (ST7)
MF5377	L. monocytogenes	Meat processing plant (ST8)
MF5378	L. monocytogenes	Meat processing plant (ST394)
MF5630	L. monocytogenes	Meat processing plant (ST19)
MF5634	L. monocytogenes	Meat processing plant (ST121)
Background microbiota		
* Listeria* spp.		
MF4030	*L. innocua*	Salmon processing plant (ST599)
MF8051	*L. innocua*	Meat processing plant (ST448)
MF2618	*L. ivanovii*	Human (CCUG 37344)
MF6987	*L. ivanovii*	Natural environment
MF2623	*L. welshimeri*	Decaying vegetation, type strain (CCUG 15529)
Non-*Listeria* strains		
MF8071	Bacillus cereus group sp.	Vegetable processing plant
MF8072	Bacillus cereus group sp.	Vegetable processing plant
MF8068	*Enterococcus* sp.	Vegetable processing plant
MF5883	*Enterococcus* sp.	Salmon processing plant
MF8070	*Leucobacter* sp.	Vegetable processing plant
MF8056	Pseudomonas sp.	Vegetable processing plant
MF6692	Pseudomonas sp.	Vegetable processing plant
MF5529	Pseudomonas sp.	Salmon processing plant
MF8054	Serratia rubidaea	Vegetable processing plant
MF8063	Staphylococcus sp.	Vegetable processing plant

a L. monocytogenes strains (34). The other strains are from the current study.

For the enrichment experiment, cultures containing the seven L. monocytogenes strains were analyzed to assess the proportion of each strain using two methods: (i) *dapE* colony PCR of colonies from *Listeria*-selective agar plates followed by Sanger sequencing (colony sequencing) and (ii) DNA extraction from culture pellets, *dapE* PCR, and Illumina MiSeq sequencing (amplicon sequencing).

**FIG 1:**
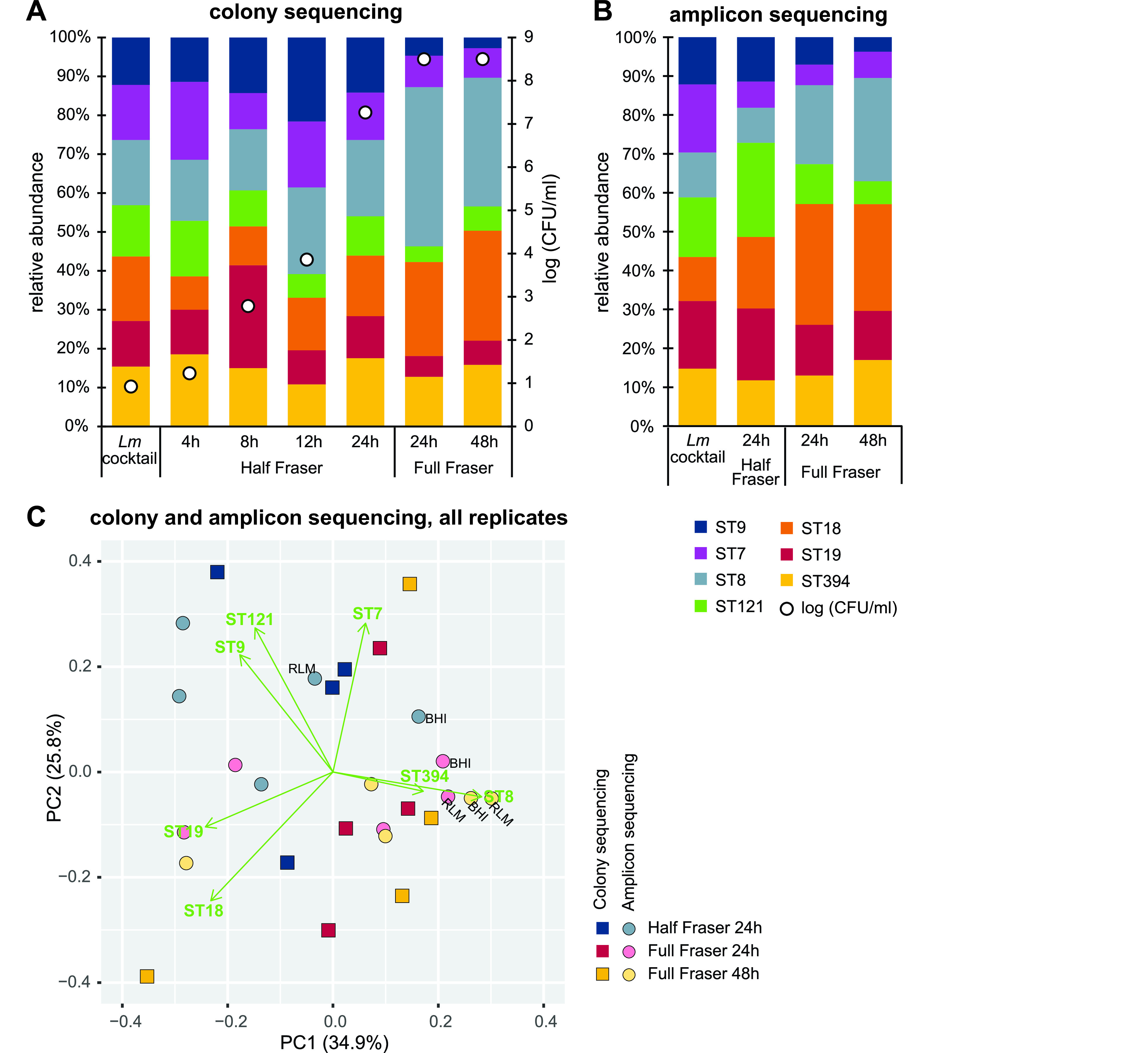
Development of the microbiota in the enrichment cultures containing seven L. monocytogenes strains. The proportion of each L. monocytogenes ST was determined by sequencing the *dapE* gene either using PCR and subsequent Sanger sequencing of individual colonies (A) or amplicon sequencing of DNA isolated from culture pellets (B). Presented results are averages from four (A) or three (B) independent experiments. Results for individual experiments are shown in Fig. S1 in the supplemental material. (C) Results from sampling points analyzed with both methods were compared using PCA.

Colony sequencing revealed all STs to be present at all analyzed time points throughout enrichment. The proportions of the individual STs fluctuated over time, with increased proportions of ST8 (significantly higher after 24 h and 48 h in Full Fraser, *P < *0.05) and ST18 (significantly higher after 24 h in Full Fraser, *P < *0.05) at later sampling points (Fig. 1A; also see Fig. S1A in the supplemental material). For amplicon sequencing, the DNA concentration for samples taken earlier than 24 h in Half Fraser was too low for successful PCR amplification of *dapE*. Attempts to increase the sensitivity of the analysis by whole-genome amplification using multiple displacement amplification (MDA) were unsuccessful. At later sampling time points, all seven STs were detected in all samples (without MDA). Increased proportions of ST8 and ST18 at the later sampling time points were observed, as also found by colony sequencing (Fig. 1B, Fig. S1B). When growth curves were established for all strains individually in Half Fraser and Full Fraser (Fig. S2), there was no statistically significant difference between the growth of the strains at any of the sampling time points between 4 h and 24 h in Half Fraser. After 24 h in Full Fraser, the concentration of ST8 was significantly higher than that of all other strains except ST18 (*P < *0.05), and the concentration of ST18 was significantly higher than that of all other strains except ST8 and ST7 (*P < *0.05). There were no statistically significant differences in the concentrations between the remaining strains at this time point, and this difference was not observed after 48 h in Full Fraser (Fig. S2). The strains that grew better when grown individually (ST8 and ST18) corresponded to those that were present in a higher abundance in the enrichment cultures. These results therefore indicate that the observed changes in the proportions of the STs in the combined L. monocytogenes enrichment culture after 24 h in Full Fraser were due to different growth capacities of the individual strains rather than complex interactions between bacteria present in the combined culture.

For the time points where results were obtained for both *dapE* colony and amplicon sequencing (Fig. 1A and B), correlation analysis indicated that the linear relationship between the proportions of individual STs determined using the two approaches was higher in Full Fraser than in Half Fraser, with correlation coefficients (Pearson’s *r*) equal to −0.17, 0.74, and 0.90 for samples analyzed after 24 h in Half Fraser, 24 h in Full Fraser, and 48 h in Full Fraser, respectively. A principal-component analysis (PCA) (Fig. 1C) showed that there was greater variation in the determined relative ratios between STs when the composition of the enrichment culture was analyzed using colony sequencing compared to amplicon sequencing. The PCA plot also showed a shift in the ratio between individual STs from 24 h in Half Fraser to the Full Fraser cultures (PC1), and that this shift was greater when the results were obtained with colony sequencing compared to amplicon sequencing. ST7, ST9, and ST121 had a higher abundance after 24 h in Half Fraser than in Full Fraser (the Half Fraser samples cluster in the first quadrant in the Fig. 1C PCA plot). There was also a tendency toward a higher proportion of ST8 in Full Fraser than Half Fraser.

To examine whether the differences between the results from colony sequencing and amplicon sequencing were due to selection during plating on *Listeria*-selective agar (RLM) or a consequence of analyzing an insufficient number of colonies for colony sequencing (numbers of analyzed colonies are indicated for each experiment in Fig. S1A), three cases were assessed. In addition to colony sequencing of individual colonies picked from RLM agar plates, samples composed of all colonies scraped off BHI and RLM agar plates were subjected to *dapE* amplicon sequencing. In all three cases, all seven STs were detected after 24 h in Half Fraser. After 24 h in Full Fraser, all STs except for ST121 were detected by colony sequencing, and after 48 h in Full Fraser, all STs except for ST19 and ST121 were detected by colony sequencing, whereas all seven STs were detected at all time points by amplicon sequencing of all colonies from the BHI and the RLM agar plate (Fig. S1C). Due to the larger variation when using colony sequencing, amplicon sequencing was used in subsequent experiments.

### Growth dynamics of enrichment cultures containing L. monocytogenes and background microbiota.

The ISO 11290-1 enrichment method for the detection of L. monocytogenes and *Listeria* spp. in the food chain (14) is not intended for use with pure cultures. For a more realistic experimental setting, mimicking a real sample from the food processing environment containing L. monocytogenes, a background microbiota community was included. Selection of candidate background microbiota strains was performed according to the criteria described in Materials and Methods, and included strains are listed in Table 1.

**FIG 2:**
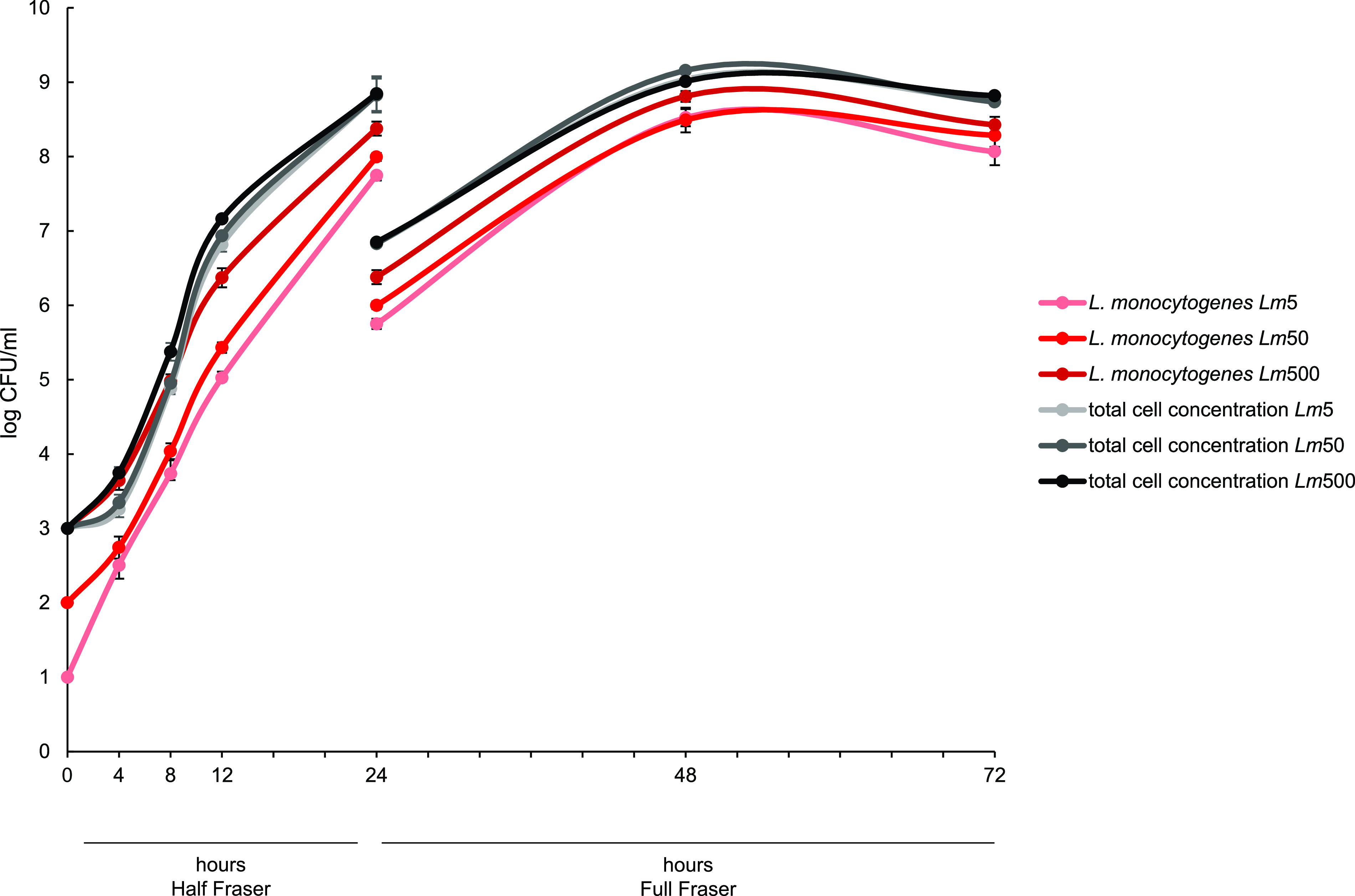
Growth curves for enrichment cultures containing L. monocytogenes and background microbiota. L. monocytogenes concentrations were evaluated by counting colonies on *Listeria*-selective agar (RLM), while total cell concentrations were evaluated by counting colonies on nonselective agar (TSA). Average values for three experiments are shown. Error bars denote the standard deviations.

To examine the growth and population dynamics of the microbiota, an enrichment experiment according to ISO 11290-1 was set up containing all seven L. monocytogenes and background microbiota strains. To determine whether the relative amount of L. monocytogenes in the enrichment culture would affect the results, three different inoculum concentrations of the L. monocytogenes cocktail (*Lm* cocktail; comprising equal ratios of all seven L. monocytogenes strains) were selected, while the concentration of the background microbiota cocktail (comprising equal ratios of all strains of the background microbiota community) was kept constant. The three mixed microbiota enrichment cultures, referred to as *Lm*5, *Lm*50, and *Lm*500, respectively, contained 1%, 9%, and 50% L. monocytogenes at the time of inoculation in Half Fraser. Growth curves established during enrichment showed that the total cell concentration differed among the three cultures, in accordance with the inoculum concentrations in Half Fraser but not in Full Fraser (Fig. 2). After 4 h and 8 h of enrichment, the total cell concentration was significantly lower in *Lm*5 and *Lm*50 than *Lm*500 (*P < *0.05). After 12 h, the total cell concentration in the *Lm*5 culture was significantly lower than that in *Lm*500 (*P < *0.05), while there was no significant difference between *Lm*50 and *Lm*500. There were no significant differences between the total cell concentrations of the three enrichment cultures at subsequent time points in Half and Full Fraser.

The concentration of L. monocytogenes also differed in accordance with the inoculum concentrations of the enrichment cultures. After 4 h, the concentrations in *Lm*5 and *Lm*50 were significantly lower than that in *Lm*500 (*P < *0.05). There was a significant difference between the L. monocytogenes concentrations in all three enrichment cultures after 8 h, 12 h, and 24 h in Half Fraser (*P < *0.05). After 24 h in Full Fraser, the L. monocytogenes concentration in *Lm*5 and *Lm*50 again differed significantly from that of *Lm*500 (*P < *0.05), but there were no significant differences after 48 h in Full Fraser.

### Detection of L. monocytogenes using a commercial qPCR kit.

A commercial qPCR kit, validated against ISO 11290-1 according to the ISO 16140 protocol (15), was used for detection of L. monocytogenes and *Listeria* spp. in all samples collected from the enrichment cultures containing L. monocytogenes and background microbiota. Neither L. monocytogenes nor *Listeria* spp. could be detected in the 4-h samples, although all samples contained more than 100 CFU/ml L. monocytogenes (Fig. 2). In the samples taken after 8 h of enrichment, L. monocytogenes and/or *Listeria* spp. were detected exclusively in the *Lm*500 cultures containing the highest concentration of L. monocytogenes (about 10^5^ CFU/ml) (Fig. 2). After 12 h of enrichment, both L. monocytogenes and *Listeria* spp. or only *Listeria* spp. were detected in the *Lm*5 cultures, while the *Lm*50 and *Lm*500 cultures were positive for both (Table S1). For all the subsequent time points, the quantification was in accordance with the plate count, as no significant differences were detected (Table S2) (paired Student's *t* test, *P > *0.05 for all samples and time points). The consistent detection of L. monocytogenes and *Listeria* spp. in all samples, even the ones with the lowest L. monocytogenes concentration, taken after 24 h, implies quicker results, corresponding to the validation of this method according to ISO 16140 compared to conducting the entire enrichment protocol. The results additionally indicate that it is possible to validate the use of commercial qPCR kits earlier than 24 h during enrichment.

### Population dynamics assessed by *dapE* amplicon sequencing, 16S rRNA amplicon sequencing, and Nanopore Flongle quasimetagenomic shotgun sequencing.

**FIG 3:**
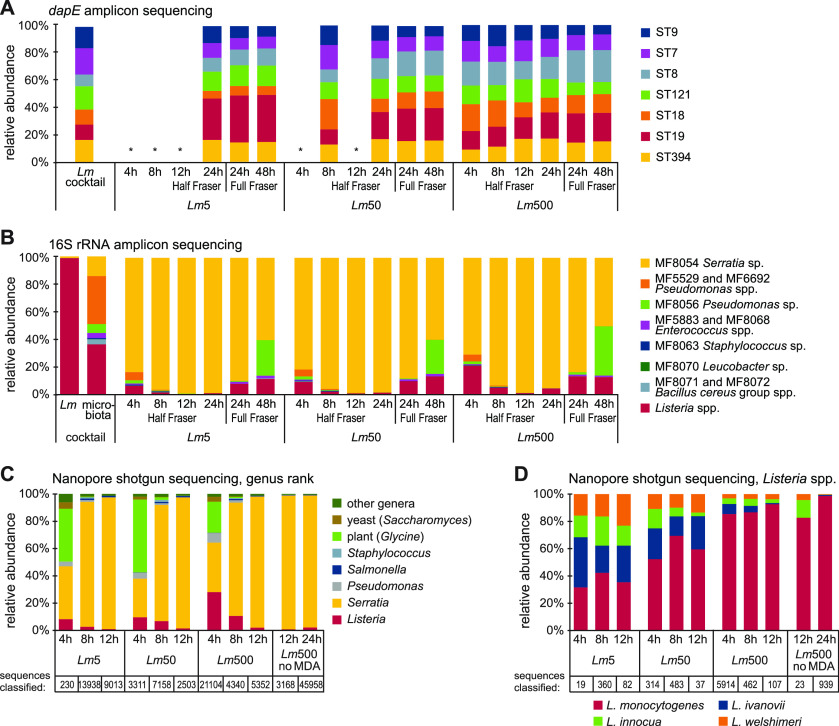
Population dynamics in the enrichment cultures containing L. monocytogenes and background microbiota. The cultures labeled *Lm*5, *Lm*50, and *Lm*500 contained 1%, 9%, and 50% of L. monocytogenes, respectively, at the time of inoculation. (A) The proportion of each L. monocytogenes ST was determined using *dapE* amplicon sequencing. The *Lm* cocktail sample constituted a mixture of all seven L. monocytogenes cultures in equal proportions. The 4-h, 8-h, and 12-h samples were subjected to whole-genome MDA prior to *dapE* amplicon sequencing. Samples labeled with an asterisk did not yield results due to insufficient DNA concentrations. Presented results are averages from three independent experiments, except the *Lm*50 8-h sample, for which results were obtained for two replicates. Results for individual experiments are shown in Fig. S4. (B) The proportion of each species was determined using 16S rRNA amplicon sequencing. The microbiota cocktail sample constituted a mixture of all L. monocytogenes and background microbiota cultures in equal proportions. Presented results are averages from three independent experiments. Results for individual experiments are shown in Fig. S5. (C and D) Taxonomic assignment of reads from Nanopore Flongle sequencing, showing genus-level classification (C) and species-level classification (D) within the *Listeria* genus. The tables at the bottom of panels C and D show the total number of classified reads in each sample.

To monitor the population dynamics in the three mixed microbiota enrichment cultures (*Lm*5, *Lm*50, and *Lm*500), *dapE* and 16S rRNA amplicon sequencing was performed to determine the relative proportions of the seven L. monocytogenes STs and each species or genus, respectively. As the DNA concentrations were not sufficient for direct *dapE* amplicon PCR for the samples taken after 4 h, 8 h, and 12 h (with the exception of two of the three replicates from the *Lm*500 cultures), whole-genome MDA was performed prior to PCR and sequencing for the samples from these time points. The MDA approach was successful for the cultures containing the highest L. monocytogenes inoculum concentration (*Lm*500) at all three early sampling time points but did not increase the DNA concentration sufficiently for the cultures with lower L. monocytogenes inoculum concentrations, with the exception of *Lm*50 after 8 h (in two of the three replicates). To examine whether MDA introduced a bias with respect to the relative abundance of each ST, the *dapE* sequencing results were compared with and without MDA for the two 12-h *Lm*500 samples (Fig. S3). The relative abundance of each ST was highly similar. The overall results showed that all seven STs were detected in different proportions (≥5.7%) at all analyzed time points (Fig. 3A, Fig. S4).

16S rRNA amplicon sequencing (Fig. 3B, Fig. S5) was successful without MDA for samples from all time points and showed that *Listeria* spp. were detected at the earliest sampling time point (after 4 h in Half Fraser) and that the relative abundance of *Listeria* spp. differed between the *Lm*5, *Lm*50, and *Lm*500 cultures, with 6, 9, and 21% (average from three independent experiments), respectively. The relative abundance of *Listeria* spp. was reduced after 8 h (1.5, 2.1, and 5%) and 12 h (0.3, 0.6, and 1%) in Half Fraser but then increased again after 24 h (1, 1.4, and 4.4%) in Half Fraser and further after 24 h (7.8, 9.8, and 13.3%) and 48 h (11.4, 13.2, and 12.7%) in Full Fraser (numbers in parentheses refer to the *Lm*5, *Lm*50, and *Lm*500 cultures, respectively). Thus, this phenomenon was observed independently of the L. monocytogenes inoculum concentration. Regarding the background microbiota diversity, the Serratia rubidaea strain dominated the cultures already after 4 h of growth in Half Fraser and at all subsequent sampling time points in all three enrichment cultures. The overall microbiota diversity decreased over time in Half Fraser but subsequently increased again after 24 h and 48 h in Full Fraser, mainly due to an increase in the relative abundance of *Listeria* spp. and Pseudomonas spp.

A possible analysis method that may facilitate quicker detection of L. monocytogenes and *Listeria* spp. and allow an estimation of the diversity in an enriched sample is quasimetagenomic shotgun sequencing. For this purpose, Nanopore Flongle sequencing was used as a quick, real-time, and cost-effective method that has the potential for on-site application in the food industry. From the first replicate of the experiment, DNA from the samples taken after 4 h, 8 h, and 12 h from all three enrichment cultures (*Lm*5, *Lm*50, and *Lm*500) was subjected to MDA and subsequently sequenced (Fig. 3C and D; quality metrics are provided in Table S3). In addition, the DNA of the samples taken after 12 h and 24 h from the *Lm*500 culture was directly sequenced without prior MDA. *Listeria* spp. were detected with the highest relative abundances in the samples after 4 h of enrichment, followed by a decrease over the subsequent two time points in all three enrichment cultures (Fig. 3C). A substantial number of reads was assigned to yeast and plants, most likely derived from the culture medium that contains esculin (a fluorescent cumarine derived from horse chestnut) and yeast extract. It is likely that this proportion could have been reduced if cell pellets had been washed prior to DNA extraction. The relative abundance of Serratia rubidaea was high in all samples, in accordance with the results obtained from the 16S rRNA amplicon sequencing (Fig. 3B). The sequences assigned to *Listeria* spp. were further classified to species level, and, as expected, an increase in the relative abundance of L. monocytogenes was observed with increasing inoculum concentration in the mixed microbiota enrichment cultures (Fig. 3D). Therefore, quasimetagenomic shotgun sequencing is, well suited to differentiate between pathogenic and nonpathogenic *Listeria* spp.

When examining the results for the single sample (*Lm*500 at 12 h) sequenced both prior to and after MDA, MDA did not appear to introduce a bias, as the relative abundance of genera/species was similar. More Nanopore sequencing data were obtained for the MDA sample (Fig. 3C and D). The lack of bias with MDA is consistent with the observed results for *dapE* amplicon sequencing of the 12-h samples with and without MDA (Fig. S3).

### Analysis of MLST alleles detected in the Nanopore sequencing data.

The enrichment cultures examined in the current study contained seven L. monocytogenes strains, selected as a diverse set that could be differentiated by their *dapE* alleles. For the six remaining MLST gene loci, the numbers of differing alleles among the seven isolates was three (*abcZ*, *bglA*), seven (*cat*), five (*dat*), six (*ldh*), or two (*ldkA*). The allele numbers and profiles for the strains are specified in Table S4. The combined output of the Nanopore Flongle sequencing efforts was mapped to the Institute Pasteur’s L. monocytogenes MLST database using the KMA (*k*-mer alignment) method (35). The following alleles, expected to be present in the cultures containing the seven different L. monocytogenes STs, resulted in single-hit matches to the sequencing data, although each allele identification was flagged as uncertain or incomplete: *bglA-5*, *dapE-6*, *dapE-7*, *ldh-4*, and *ldh*-*37*. In addition, matches were made to four alleles that were not expected to give a positive match: one hit each for the loci *bglA*, *dapE*, *dat*, and *lhkA*. Although a match to two different expected (and one unexpected) *dapE* alleles was obtained, this certainly does not constitute reliable identification of more than one ST. However, considering that only 8,741 Nanopore reads were mapped to *Listeria* spp. in the taxonomic classification (Fig. 3D, comprising a total of 12.6 Mb, with 7,195 reads classified to L. monocytogenes), that the genome size of L. monocytogenes is 3 Mb, and that the error rate of Nanopore reads is between approximately 5 and 15% (36, 37), the outcome was roughly as expected.

### IMS and amplicon sequencing on early enrichment samples.

**FIG 4:**
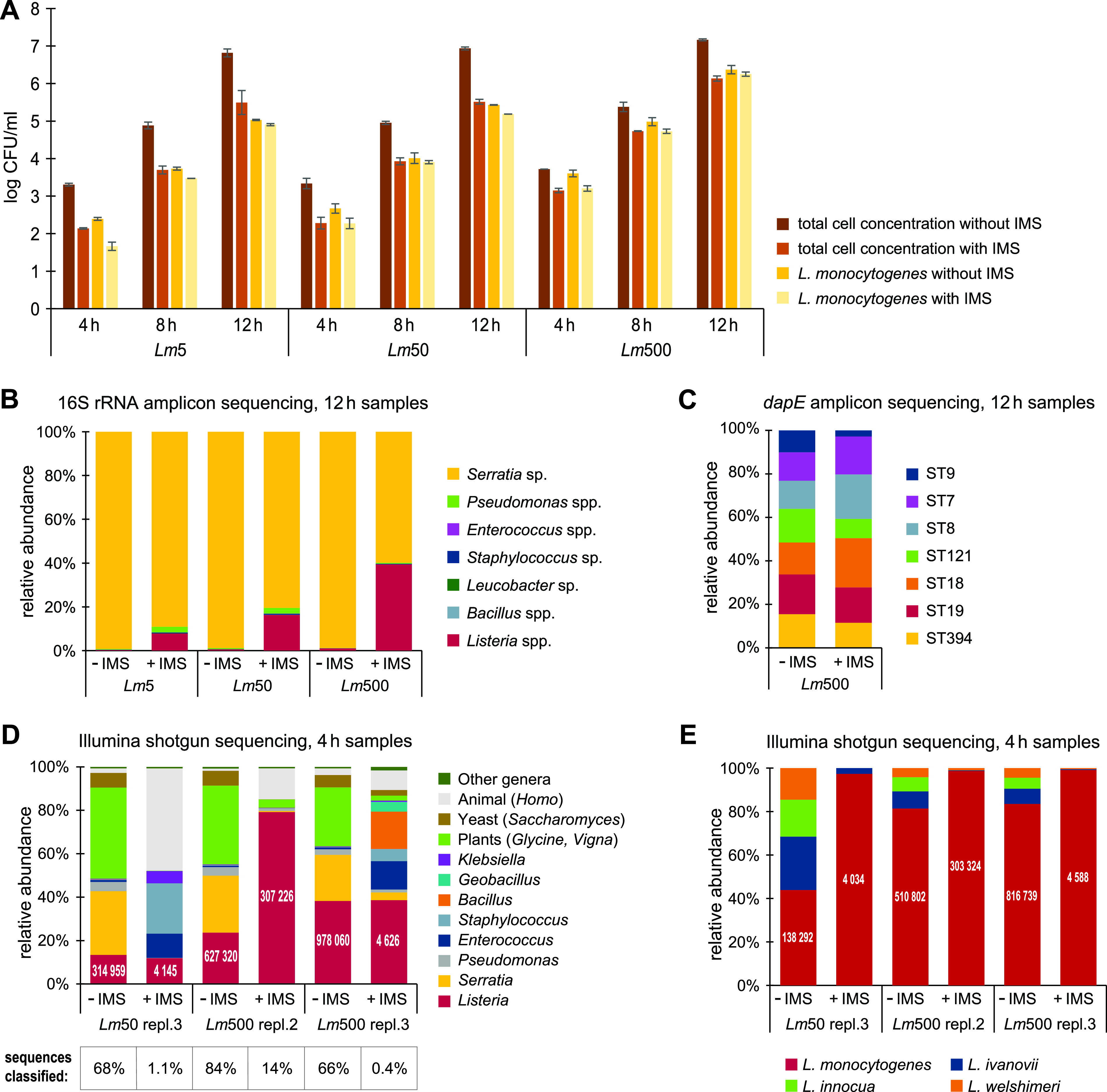
Effect of IMS. Samples were collected from Half Fraser enrichment cultures and the experiment was performed twice (replicates 2 and 3). (A) Comparison of total cell concentrations and L. monocytogenes concentrations without and with IMS. Mean values are shown, and error bars show the standard deviations. (B and C) Average relative abundances of the microbiota in 12-h samples determined by amplicon sequencing targeting the 16S rRNA (B) and the *dapE* gene (C). (D and E) Taxonomic assignment of reads from Illumina MiSeq quasimetagenomic shotgun sequencing of 4-h samples, showing genus-level classification (D) and species-level classification (E) within the *Listeria* genus. The table in panel D shows the percentage of classified reads in each sample, and the numbers printed on the columns indicate the actual number of reads classified as *Listeria* spp. (D) or L. monocytogenes (E).

Immunomagnetic separation (IMS), involving the use of magnetic beads with covalently attached antibodies specific against *Listeria*, was performed for samples collected after 4 h, 8 h, and 12 h of growth in Half Fraser. All three cultures containing different concentrations of L. monocytogenes relative to the background microbiota (*Lm*5, *Lm*50, and *Lm*500) were included. One wash step was used prior to elution, as determined to be optimal in preliminary experiments (data not shown). The concentration of L. monocytogenes and the total cell concentration were lower in the samples subjected to IMS than in the samples without IMS. However, the log CFU/ml reduction was significantly higher for the total cell concentration (1.05 log CFU/ml [standard errors [SE], ±0.07]) than for the L. monocytogenes concentration (0.29 log CFU/ml [SE, ±0.05]) (paired Student's *t* test, *P < *0.05, calculated on log reduction values across time points and enrichment cultures) (Fig. 4A), indicating that the IMS procedure was able to enrich for L. monocytogenes cells relative to the background microbiota during enrichment.

The composition of the microbiota was analyzed and compared with and without IMS using 16S rRNA amplicon sequencing. While 16S rRNA sequencing was successful for the samples without IMS from all time points (Fig. 3B), amplification of 16S rRNA was not successful on samples with IMS from after 4 h and 8 h of enrichment. MDA was attempted for these samples but did not increase the DNA concentration sufficiently for successful 16S rRNA amplification. Therefore, only the samples taken after 12 h were analyzed (Fig. 4B). While Serratia rubidaea showed the highest relative abundance regardless of IMS, the relative abundance of *Listeria* spp. increased with IMS and with increasing L. monocytogenes inoculum concentration (from 0.3 to 7.8% in *Lm*5, from 0.6% to 16% in *Lm*50, and from 1% to 39% in *Lm*500). In addition to *Listeria* spp., Pseudomonas spp. increased in relative abundance after IMS, but this effect was more pronounced in the *Lm*5 and *Lm*50 cultures than in the *Lm*500 culture. These results do not provide any conclusions regarding the proportion of L. monocytogenes within the relative *Listeria* species abundance, since the species are not distinguishable by partial sequencing of the 16S rRNA gene.

To determine the proportions of the seven L. monocytogenes STs in the enrichment cultures with and without IMS, *dapE* amplicon sequencing was performed. The DNA concentration was too low for successful *dapE* PCR (also after MDA) for all samples subjected to IMS after 4 h and 8 h of enrichment and for the *Lm*5 and *Lm*50 cultures harvested after 12 h. Therefore, only the samples from the *Lm*500 cultures harvested after 12 h of enrichment with IMS were subjected to *dapE* sequencing (following MDA). Similar to what was observed in samples not subjected to IMS (Fig. 3A), all seven L. monocytogenes strains were detected in samples subjected to IMS (Fig. 4C). While the proportions of ST9, ST19, ST121, and ST394 decreased after the application of IMS (from 10% to 3%, 18% to 16%, 15% to 9%, and 15% to 12%, respectively), the proportions of ST7, ST8, and ST18 increased (from 13% to 17%, 13% to 20%, and 15% to 23%, respectively) (Fig. 4C).

### Sample diversity assessed by Illumina MiSeq quasimetagenomic shotgun sequencing.

The use of Nanopore Flongle flow cells for quasimetagenomic shotgun sequencing enabled the detection of L. monocytogenes as well as differentiation between the pathogenic L. monocytogenes and nonpathogenic *Listeria* spp. already after 4 h of enrichment (Fig. 3D). A prediction about cooccurring STs could not be obtained, potentially due to low sequencing coverage and the high error rates currently inherent to the Nanopore sequencing technology. To examine whether it would be possible to detect and identify cooccurring STs from the early enrichment cultures with a more efficient (although more time-consuming) sequencing technology, selected samples collected after 4 h of enrichment in Half Fraser were subjected to quasimetagenomic shotgun sequencing on an Illumina MiSeq. In addition, as 16S rRNA and *dapE* amplicon sequencing had been unsuccessful following IMS for samples collected early during enrichment, we also subjected the corresponding IMS samples to Illumina MiSeq shotgun sequencing to assess the effect of IMS during an early stage of enrichment. The selected samples involved DNA subjected to MDA from the *Lm*50 culture from the third replicate and the *Lm*500 cultures from the second and third replicate of the experiment with and without IMS. The number of paired-end reads obtained for the six samples ranged from 2.8 to 3.8 Mb.

As with quasimetagenomic shotgun sequencing performed using Nanopore technology (Fig. 3D and E), Illumina sequencing was able to detect dominating microbiota (Fig. 4D) and differentiate between different *Listeria* species (Fig. 4E) in all samples collected after 4 h of selective enrichment. Furthermore, the analysis confirmed that the relative abundance of L. monocytogenes was higher after 4 h than after 12 h of enrichment in Half Fraser. The output of the Illumina sequencing was filtered using Kraken to only contain reads classified as *Listeria* spp. or L. monocytogenes (1.8 million 2×300-bp paired-end sequencing reads; giving in total over 1 Gb) and mapped to the Institute Pasteur’s L. monocytogenes MLST database using the KMA (*k*-mer alignment) method (35). This analysis resulted in detection of 28 out of the 33 expected alleles (Table S4), missing *cat*-*2* from MF5377 (ST8), *dapE*-*21* from MF5378 (ST394), *dat-5* and *dat*-*6* from MF5376, MF5377, and MF5634 (ST7, ST8, and ST121, respectively), and *ldh*-*4* from MF4536 (ST9). The detailed analysis output presented in Table S5 shows that in general, higher scores were obtained for expected alleles but also that a large number of false-negative allele calls were reported. This is to be expected with short read sequencing data (reads are 300 bp in length and alleles range in size from 399 to 537 bp), since the ConClave scoring scheme used in KMA (35) adds the alignment score to all matching alleles if a single read matches equally well to more than one allele in the database. Although this analysis was not able to (nor designed for) detection of specific STs in mixed-culture samples, it clearly predicts that multiple strains of L. monocytogenes are present.

The application of IMS led to a reduction in the amount of sequenced DNA originating from plants and yeast from between 33 and 49% of classified reads without IMS to between 0.1 and 5% with IMS (Fig. 4D), likely due to the washing step included in the IMS procedure. However (and in contrast to the data obtained with Nanopore sequencing), reads classified as Homo sapiens comprised a substantial amount of Illumina sequences, particularly in samples subjected to IMS (up to 47%). These largely constituted highly repetitive sequences that are likely not specific to H. sapiens, as this species was the only mammalian species included in the database used for taxonomic classification. The large number of repetitive sequences and the much smaller proportion of classified reads obtained for samples subjected to IMS relative to samples not subjected to IMS (see the table at the bottom of Fig. 4D) illustrate the increasing challenge inherent to metagenomic shotgun sequencing for samples with decreasing biomass.

Nevertheless, the analysis could confirm that the application of IMS clearly increased the level of L. monocytogenes relative to other *Listeria* spp. (Fig. 4E). The increase in relative abundance of Pseudomonas spp. in the samples subjected to IMS observed with 16S rRNA amplicon sequencing after 12 h of enrichment (Fig. 4B) was not paralleled in the data obtained from quasimetagenomic shotgun sequencing of the 4-h samples (Fig. 4D). After 4 h, IMS instead increased the proportion of strains from Gram-positive genera (*Bacillus*, Staphylococcus, and *Enterococcus*) and almost completely eliminated the presence of the Gram-negative Serratia rubidaea strain from between 21 and 29% without IMS to between no reads and 4% with IMS.

## DISCUSSION

The idea of using quasimetagenomic sequencing for early pathogen detection has been pursued by FDA scientists since 2009 in efforts to recover pathogens from complex microbiomes (29, 38–44). A recent study by Ottesen et al. (26) showed that the application of quasimetagenomics to detect L. monocytogenes in naturally contaminated ice cream was successful after 20 h of enrichment. Phylogenetic source tracking was possible using sequencing data obtained from enrichment cultures from 24 h onwards to 48 h of enrichment, giving the same result as WGS data from individual isolates obtained by classical culture techniques. Despite the substantial reduction of time from sampling to source tracking, there are still many unanswered questions regarding the effect of enrichment on L. monocytogenes population dynamics, including whether quasimetagenomic sequencing is unconditionally suitable as a tool for early detection and source tracking of L. monocytogenes in the food industry. There have been concerns that the current standard workflow for strain typing selects for certain L. monocytogenes STs during enrichment or subsequent plating (23–25). Our results indicate that there is little risk that the enrichment process will select for or lead to the loss of specific L. monocytogenes STs commonly isolated from the food industry. It should be noted, however, that this study did not aim to investigate the differences between different genetic subtypes but focused on seven STs commonly found in the food industry. Given that all seven strains coenriched in this study were recovered at all selected time points throughout enrichment (4 h, 8 h, 12 h, and 24 h in Half Fraser and 24 h and 48 h in Full Fraser), the WGS workflow used by the food industry would randomly identify only one of the strains, as usually a single colony is picked from a plate at the end of the enrichment procedure. To detect cooccurring strains, it is necessary to either identify several colonies or use (quasi)metagenomic sequencing. Similar results for proportions of the STs were obtained from both *dapE* colony sequencing and amplicon sequencing, although colony sequencing resulted in a larger variation between trials and time points. This is to be expected considering the relatively low number of colonies sequenced per time point compared to the great number of sequences obtained by amplicon sequencing. One drawback of amplicon sequencing, however, was the low sensitivity of the *dapE* PCR, resulting in unsuccessful amplification from samples collected earlier than 24 h of enrichment in Half Fraser when the initial L. monocytogenes load was low. The *dapE* gene was successfully used to discriminate between the seven STs in the current study, but *dapE* does not discriminate between all known STs, and the use of other target genes may be needed in other situations. MDA was tested prior to *dapE* amplicon sequencing but was not always successful. *Listeria* only has one copy of the *dapE* gene in the genome, and MDA is troubled with amplification bias, which results in a different amplification coverage across the genome (45–47). MDA prior to *dapE* amplicon sequencing and quasimetagenomic shotgun sequencing was essential to achieve enough DNA from the low biomass samples and did not seem to introduce any bias compared to the samples not subjected to MDA.

In a previous study, culture enrichment time prior to shotgun metagenomics of Salmonella was substantially shortened by IMS followed by MDA (28). IMS enabled the selective capture of Salmonella cells before they reached high abundances in the enrichment culture, and subsequent MDA generated sufficient genomic DNA for shotgun sequencing already after 4 h of enrichment. In the current study, IMS enabled selective capture of *Listeria* spp. and reduced the levels of inoculated competitive bacteria. Unfortunately, it also reduced the total number of *Listeria* spp. and L. monocytogenes sequences. Low recovery rates of approximately 1 to 2 log below the initial inoculum level, after the application of anti-*Listeria* Dynabeads, have also previously been reported (48–50). These results indicate that the application of IMS is not ideally suited for recovery of L. monocytogenes early in the enrichment when the expected biomass is low, as the risk of losing L. monocytogenes might have a greater negative impact on downstream analyses than the obtained relative enrichment.

To shorten the time from collecting a sample to detecting L. monocytogenes, validated methods employing qPCR after 24 h of primary enrichment have been established (51–53). In this study, L. monocytogenes was detected at earlier time points than 24 h during primary enrichment. While these results suggest a possible validation of the use of commercial qPCR kits earlier than 24 h during enrichment, the reduction of the detection time to 24 h is already a substantial improvement since an additional 24 h of enrichment are saved. In order to generate genetic information, however, subsequent genetic typing using a method such as MLST, or, preferably, WGS, must be performed.

WGS is predominantly applied in outbreak situations by food authorities or researchers but is becoming more important as a surveillance tool for source tracking of pathogens in the food industry (16, 54). While other studies have shown the potential of Illumina MiSeq and Oxford Nanopore MinION sequencing (26, 28, 29), we wanted to test Nanopore Flongle flow cells as a low-cost, real-time sequencing alternative. Despite the yield being lower than that of MinION flow cells, we showed Flongle flow cell sequencing to be a tool with potential for on-site application in the industry in combination with short enrichment for early detection of L. monocytogenes and for estimating the identity of the total microbiota in a sample. Nanopore Flongle and Illumina MiSeq quasimetagenomic shotgun sequencing yielded very similar results for the 4 h samples with respect to the identity of the total microbiota and the abundance of *Listeria* spp. and L. monocytogenes, indicating that Nanopore Flongle is an affordable alternative for the food industry for quality surveillance.

The duration of culture enrichment required to achieve a certain limit of detection will depend on several factors, such as the total microbiota of the sample and growth dynamics of target organisms as well as the food matrix. In the present study, we chose a background microbiota community that was selected based on frequent presence in different food industry sectors and the ability to grow well in the enrichment media. This resulted in dominance of the Serratia rubidaea strain throughout the enrichment. This contrasts with the results obtained by Ottesen et al. (55), who found that L. monocytogenes dominated in naturally contaminated ice cream after 24 h of enrichment in three different enrichment media, including the same medium as that used in the current study. However, they also found that L. monocytogenes was proportionally greater at hour 0 than when tested after 4 h, 8 h, and 12 h, indicating potential competitive exclusion of L. monocytogenes by *Anoxybacillus* and *Geobacillus* in early enrichment hours (26, 55). In our experiments, the tested samples with the highest relative amount of L. monocytogenes were collected after 4 h of enrichment. Whether L. monocytogenes would have had a higher relative value in the presence of other background microbiota is not known, but our results and those of others (26, 55) clearly show that in the presence of certain background microbiota strains, L. monocytogenes will not dominate even after several hours of enrichment in selective medium. This reflects the balance between an optimal recovery capacity for stressed L. monocytogenes cells and the suppression of background microbiota when choosing an enrichment broth.

Although we did not achieve the expected output of data for the Nanopore Flongle sequencing, we were still able to separate the *Listeria* sequences into the four different added species, including L. monocytogenes. The data obtained by Flongle sequencing also enabled the analysis of the total microbiota, data that could be useful for the food industry in their quality surveillance. For example, a recent study using high-throughput full-length 16S rRNA gene sequencing to investigate the presence of *Listeria* spp. in a meat processing plant in the context of whole bacterial communities found correlation patterns regarding the presence/absence of *Listeria* spp. (56). There is still much work to be done regarding the use of the core in-house processing microbiome as an indicator for pathogen presence, but monitoring of the food industry environmental microbiome is a promising tool that could support overall quality and safety management plans (57).

With respect to the question of determining whether cooccurring L. monocytogenes STs are present in a sample, our results showed that this cannot be predicted from the limited amount of data obtained in this study using Nanopore Flongle shotgun sequencing. The data obtained from Illumina MiSeq shotgun sequencing did allow us to predict cooccurring STs in the sample but not the exact number of STs present. However, the performed *in silico* MLST analysis is designed for analysis of single isolates rather than metagenomes, and, to our knowledge, a program for prediction of STs from (quasi)metagenomic shotgun sequencing data is currently not available. A recent study evaluated the accuracy of L. monocytogenes assemblies from enrichments (quasimetagenomes) of naturally contaminated ice cream using long-read (Oxford Nanopore GridION) and short-read (Illumina MiSeq) sequencing data (29). The study showed that it was possible to reconstruct a circularized genome as well as a plasmid from Nanopore long-read sequencing data obtained after 24 h of enrichment but that the high error rates prevented high-fidelity gene assembly, even at high depth of coverage. Illumina short-read assemblies accurately reconstructed the core genes after 24 h of enrichment but produced highly fragmented genomes. Based on their results, the authors proposed a hybrid approach. We did not attempt to reconstruct any genomes or plasmids with our data, as our aim was primarily to detect L. monocytogenes and to predict cooccurring STs. However, due to the low coverage of long-read data obtained in the current study, we did not expect that successful reconstruction would be possible. Nevertheless, we showed that Nanopore Flongle quasimetagenomic shotgun sequencing can be an affordable tool for the food industry for routine quality surveillance. Although it was not able to predict cooccurring L. monocytogenes strains, it provided fast, real-time, and valuable information after just 4 h of culture enrichment, allowing results to be obtained in less than 24 h after sampling. Illumina MiSeq quasimetagenomic shotgun sequencing provided clear indication of cooccurring L. monocytogenes strains after 4 h of enrichment, and even though it is more time-consuming and expensive, it could be a valuable tool for occasional testing in the food industry.

In conclusion, we showed that the culture enrichment can introduce a bias between cooccurring L. monocytogenes strains after 24 h in Half Fraser when different L. monocytogenes STs are enriched without background microbiota, but this was less apparent when cocultured with background microbiota. We also showed that colony and amplicon sequencing provided similar results, but, as expected, colony sequencing resulted in more variation due to the smaller amounts of sequences compared to amplicon sequencing. For faster results, we demonstrated the potential of Nanopore Flongle sequencing as an affordable, real-time approach for the analysis of both the total microbiota and the presence of L. monocytogenes after just 4 h of culture enrichment. It is important to acknowledge that this analysis is neither quantitative nor able to discriminate between live/dead bacteria. Still, it could provide fast and useful additional information for the food industry. For the investigation of cooccurring L. monocytogenes strains, it would be ideal if the analysis applied in the food industry involved sequencing and subsequent typing of several colonies picked from selective agar plates or performing quasimetagenomic sequencing within the first 24 h of culture enrichment.

## MATERIALS AND METHODS

### Bacterial strains.

The bacterial strains used in this study are listed in Table 1. Seven L. monocytogenes strains from three different meat processing facilities were selected (58) based on previously described selection criteria (34). All seven selected strains had different *dapE* MLST alleles (59), enabling their differentiation by sequencing. The selected background microbiota strains were from genera commonly isolated from food processing environments (60). We initially selected 71 strains from our strain collection of genera/species shown dominant in previous studies in the following processing environments: cheese (61), meat (62), salmon (63), and fresh produce (64). These 71 strains were tested for growth in Half Fraser at 30°C in a Bioscreen C instrument (Oy Growth Curves). Ten of these strains grew to an optical density at 600 nm (OD_600_) of >0.2 after 24 h and were selected for inclusion in the current work: Pseudomonas spp. (*n* = 3), *Enterococcus* spp. (*n* = 2), Bacillus cereus group species (*n* = 2), Serratia rubidaea (*n* = 1), Staphylococcus sp. (*n* = 1), and *Leucobacter* sp. (*n* = 1). For all 10 selected strains, growth was also confirmed in Full Fraser (Bioscreen C, OD_600_ of >0.5 after 48 h at 37°C). In addition, five non-*monocytogenes Listeria* strains were included, Listeria innocua (*n* = 2), Listeria ivanovii (*n* = 2), and Listeria welshimeri (*n* = 1), leading to inclusion of a total of 15 background microbiota strains in the current work (Table 1). Prior to all experiments, bacteria from stock cultures (stored at −80°C in 20% glycerol) were plated on tryptic soy agar (TSA) and grown for 48 h at 30°C.

### Growth and population dynamics of L. monocytogenes strains during culture enrichment.

Single colonies of the seven L. monocytogenes strains were each inoculated in 5 ml tryptic soy broth (TSB) and grown overnight at 30°C shaking at 150 rpm. The overnight cultures had a concentration of approximately 10^9^ CFU/ml. The cultures were mixed in equal ratios (*Lm* cocktail) and serially diluted 1:10 in peptone water and (for the last three dilution steps) in Half Fraser (dissolved and autoclaved Fraser broth base supplemented with Half Fraser supplement; Oxoid) to 10 CFU/ml. Dilutions were plated on *Listeria*-selective RAPID *L. mono* (RLM; Bio-Rad) agar plates to confirm the inoculum concentration. To determine the relative abundances of the individual strains, 2 ml of the *Lm* cocktail was centrifuged at 13,000 rpm for 5 min, the supernatant was removed, and the pellet was stored at −20°C for subsequent DNA isolation and sequencing. The 10 CFU/ml dilution was used for enrichment according to ISO 11290-1 (14). Depending on the sampling time point, different cultivation volumes were used to ensure enough biological material for downstream analysis: two volumes of 5 ml were prepared for each mix for the 4-h and 8-h time points, and one volume of 5 ml was prepared for all other time points. The cultures were first incubated in Half Fraser at 30°C for 24 h. After 24 h, they were diluted 1:100 in 10 ml Full Fraser (dissolved and autoclaved Fraser broth base supplemented with Full Fraser supplement; Oxoid) and incubated at 37°C for 48 h. After 4 h, 8 h, 12 h, and 24 h in Half Fraser and after 24 h and 48 h in Full Fraser, samples were diluted and plated on RLM agar plates to determine cell counts. In parallel, samples were harvested by centrifugation at 13,000 rpm for 5 min at 4°C. The following volumes were harvested: 10 ml after 4 h and 8 h, 5 ml after 12 h and 1 ml after 24 h in Half Fraser, and after 24 h and 48 h in Full Fraser. The supernatants were removed and the pellets stored at −20°C. This experimental setup was independently repeated four times. In addition, samples were diluted and plated on BHI and RLM agar plates after 24 h in Half Fraser and 24 h and 48 h in Full Fraser. The plates were flushed with 1.5 ml peptone water, and all colonies were scraped off the plates, pelleted by centrifugation, and stored at −20°C. This experimental setup was performed once.

Growth curves were established for the individual L. monocytogenes strains in Half Fraser (30°C, 24 h) and Full Fraser (1:100 dilution of Half Fraser cultures; 37°C, 48 h). Overnight cultures were prepared as before with the exception of incubation at 37°C. The cultures were serially diluted 1:10 in peptone water and (for the last three dilution steps) in Half Fraser to 100 CFU/ml. Cell counts were determined by plating on TSA. This experimental setup was independently repeated three times. Statistical analysis was performed using Minitab v.19 software. Values for the number of CFU/ml of each ST were log_10_ transformed prior to analysis and compared within each time point using one-way analysis of variance with the ST as factor. Tukey’s *post hoc* test for pairwise comparisons was performed to identify significant differences between the STs. Significant differences were reported at a *P *value of <0.05.

### Strain-specific quantification of L. monocytogenes using *dapE* colony PCR and subsequent Sanger sequencing.

The proportions of the individual L. monocytogenes strains were determined by DNA sequencing of the *dapE* MLST allele of randomly picked colonies plated on RLM agar. PCR and sequencing was performed as described by Ragon et al. (59) using the primers listed at https://bigsdb.pasteur.fr/listeria/primers_used.html. Sequencing was performed on an ABI 3500 Genetic Analyzer (Applied Biosystems). Obtained *dapE* sequences were assigned allele numbers in accordance with the L. monocytogenes MLST database (http://bigsdb.pasteur.fr/listeria/) (59). Statistical analysis was performed as described above, including log_10_ transformation of the number of colonies prior to analysis.

### Growth and population dynamics of L. monocytogenes during culture enrichment with background microbiota.

Single colonies of the seven L. monocytogenes and the 15 background microbiota strains were inoculated in 5 ml TSB overnight at 30°C with shaking at 150 rpm to a final concentration of approximately 10^9^ CFU/ml. Two strain cocktails were prepared from the overnight cultures. One included the seven L. monocytogenes strains (*Lm* cocktail), and the other one included the 15 background microbiota strains (background microbiota cocktail) in equal proportions. The *Lm* cocktail and the microbiota cocktail were subsequently serially diluted 1:10 as before to 10 CFU/ml and 1,000 CFU/ml, respectively. Three experimental mixed microbiota enrichment cultures (*Lm*5, *Lm*50, and *Lm*500) were prepared from the *Lm* and the background microbiota cocktail (1:1) at different concentrations. While the same final concentration of the background microbiota cocktail was used in all three cultures (500 CFU/ml), three different final concentrations of the *Lm* cocktail were used (5, 50, and 500 CFU/ml for *Lm*5, *Lm*50, and *Lm*500, respectively). The Half Fraser mixed microbiota enrichment cultures were incubated at 30°C for 24 h. Depending on the time point, different cultivation volumes were used to ensure enough biological material for downstream analysis: two volumes of 6 ml were prepared for each culture for the 4-h, 8-h, and 12-h time points, and one volume of 5 ml was prepared for each culture for the 24-h time point in Half Fraser. After 24 h, each enrichment culture was diluted 1:100 in 10 ml Full Fraser and incubated at 37°C for 48 h. Samples were harvested by centrifugation at 13,000 rpm for 5 min at 4°C. The following volumes were harvested: 9 ml after 4 h and 8 h, 4.5 ml after 12 h, and 1 ml after 24 h in Half Fraser as well as after 24 h and 48 h in Full Fraser. The supernatants were removed and the pellets stored at −20°C. In parallel, culture samples were diluted and plated on TSA and RLM agar plates in duplicates to determine cell counts. TSA plates were incubated at 30°C, while RLM agar plates were incubated at 37°C. To determine the relative abundances of the microbiota, all overnight cultures were mixed at equal proportions (microbiota cocktail) without further dilution. One milliliter of the microbiota cocktail and 1 ml of the *Lm* cocktail were harvested separately by centrifugation at 13,000 rpm for 5 min, the supernatants were removed, and the pellets stored at −20°C for subsequent DNA isolation and sequencing. This experimental setup was independently repeated three times.

### DNA isolation.

Harvested pellets were resuspended in 500 μl 2× Tris-EDTA (TE) buffer with 1.2% Triton X-100. Cells were lysed using lysing matrix B tubes (MP Biomedicals) and a Precellys Evolution instrument (Bertin Instruments) at 7,400 rpm for 2 rounds of 40 s, and genomic DNA was isolated using the DNeasy blood and tissue kit (Qiagen) according to the manufacturer’s instructions with the following modifications: twice the amount of supernatant, proteinase K, buffer AL, and ethanol were used prior to adding samples to each column.

### Strain-specific quantification of L. monocytogenes using *dapE* amplicon sequencing.

The *dapE* amplicon sequencing was performed according to the 16S rRNA metagenomics library preparation guide from Illumina with custom primers for *dapE*, as previously described (34). Sequencing (2 × 300 bp) was performed using the MiSeq reagent kit v3 on a MiSeq instrument (Illumina). The sequences were processed in QIIME2 (qiime2-2020.2) (65). Briefly, the demultiplexed Illumina data were imported into QIIME2 using the casava-18-paired-end-demultiplexed input path. Paired ends were joined using vsearch, quality filtered based on a q-score above 30, and dereplicated using vsearch, and a closed reference search (100% identity) was performed using custom *dapE* fasta and taxonomy files with the seven different *dapE* allele variants (66–68). The taxonomy tables were collapsed to ST level, converted to relative values, and exported to text files and further processed in Excel.

### Species-specific quantification using 16S rRNA amplicon sequencing.

16S rRNA gene PCR (V4 region) and paired- end sequencing (2 × 150 bp) using the MiSeq reagent kit v3 on a MiSeq instrument (Illumina) were performed using the protocol presented by Caporaso et al. (69) as previously described (70). The sequences were processed in QIIME2 (qiime2-2020.2) (65). Briefly, the data were demultiplexed using demux, and paired ends were joined using vsearch, quality filtered based on a q-score above 30, and denoised using deblur, and taxonomy was achieved using a closed reference database search (100% identity) with the sequences included in the microbiota cocktail (66–68, 71–73). The taxonomy tables were collapsed to MF isolate identity level, converted to relative values, and exported to text files and further processed in Excel.

### qPCR.

In an alternative method to ISO 11290-1, cultures can be analyzed using qPCR after 24 h of primary enrichment to obtain quicker results than by completing the entire primary and secondary enrichment protocol (15). Samples that yield positive qPCR results are then further analyzed by plating on selective agar to verify the result. Every commercial qPCR kit has to be validated according to the ISO 16140 standard protocol using the ISO 11290-1 standard as a reference method for the detection of L. monocytogenes and *Listeria* spp. in the food chain (74). qPCR was performed using the foodproof *Listeria* plus L. monocytogenes detection LyoKit qPCR kit (Biotecon Diagnostics) and a QuantStudio 5 real-time PCR system (96-wells, 0.2 ml wells; Applied Biosystems). This multiplex PCR kit is composed of a qPCR master mix that allows the simultaneous detection of L. monocytogenes and *Listeria* spp. in one reaction by using two different fluorescence channels. The kit is intended for qualitative assessment of *Listeria* in purified DNA from enrichment cultures with a limit of detection of 1 to 10 CFU of *Listeria* in 25 g of sample after 48 h of secondary enrichment and should be able to detect 0.1 to 1 CFU/μl in purified sample DNA (15). The kit was used according to the manufacturer’s instructions with the following modifications: instead of 25 μl of DNA, 5 μl of isolated genomic DNA plus 20 μl PCR grade water were used. The three independently performed culture enrichment experiments with background microbiota were assessed in separate qPCR assays, including standard curves from dilution series of genomic DNA isolated from the seven L. monocytogenes overnight cultures mixed in equal proportions (*Lm* cocktail). The DNA concentration of each experiment was correlated with the respective CFU count, and the DNA was serially diluted 1:10 in PCR-grade water to obtain eight standards, ranging from 1 to 10^7^ CFU/25-μl reaction mix. Each standard and each sample were measured once. Data were analyzed using QuantStudio software (Applied Biosystems) and Excel. Statistical analysis was performed in Excel. Values for the number of CFU/μl obtained by qPCR and plate counts were log_10_ transformed prior to analysis and compared using a paired Student's *t* test to determine statistical differences between the qPCR quantification and the plate count after 24 h in Half Fraser and 24 h and 48 h in Full Fraser.

### IMS.

Specific immunomagnetic separation (IMS) for *Listeria* was assessed using anti-*Listeria* Dynabeads (Applied Biosystems). Samples from the L. monocytogenes culture enrichment experiments with background microbiota harvested after 4 h, 8 h, and 12 h in Half Fraser were subjected to IMS. For each time point, IMS was performed in two approaches: the beads were washed once and then either eluted in 1 ml wash buffer, diluted and plated in duplicates on TSA and RLM agar plates to determine cell counts, or harvested by centrifugation and stored at −20°C after removal of the supernatant for subsequent DNA isolation. This experiment was performed twice (in replicate 2 and 3 of the population dynamics experiment). Log reduction was calculated by subtracting the log_10_ CFU/ml values with IMS from the log_10_ CFU/ml values without IMS. Statistical analysis was performed in Excel. A paired Student's *t* test was calculated on mean log reduction values across time points and enrichment cultures.

### MDA.

Multiple displacement amplification (MDA) was performed using the Qiagen REPLI-g UltraFast minikit (Qiagen) according to the rapid whole-genome amplification protocol (SQK-RAD004; Oxford Nanopore Technologies). In brief, 1 μl DNA was mixed with 1 μl buffer D1, vortexed, and incubated at room temperature for 3 min. Subsequently, 2 μl buffer N1 was added, and the mixture was vortexed and mixed with 15 μl REPLI-g UltraFast reaction buffer and 1 μl REPLI-g UltraFast reaction polymerase. The reaction mix was gently flicked, spun down, and incubated at 30°C until the DNA concentration was >80 ng/μl. The DNA concentration was quantified using a Qubit dsDNA BR assay kit (Invitrogen). The polymerase was inactivated by heating the sample at 65°C for 3 min, and the amplified DNA was stored at −20°C until required.

### Quasimetagenomic shotgun sequencing on Nanopore Flongle flow cells.

Selected samples from the first replicate of the L. monocytogenes culture enrichment experiment with background microbiota harvested at all time points throughout enrichment in Half Fraser were subjected to quasimetagenomic shotgun sequencing on Flongle flow cells. After MDA and subsequent quantification of DNA using Qubit, libraries were prepared separately for each selected sample using the SQK-RAD004 rapid sequencing kit (Oxford Nanopore Technologies) according to the manufacturer’s instructions, using 3.75 μl of 200 ng DNA as input. Each sample was sequenced on separate FLO-FLG001 Flongle flow cells in a MinION Mk1 sequencing device with MinKNOW UI 4.0.20 software. Reads in fast5 format obtained from MinKNOW were basecalled using guppy v4.2.2 (Oxford Nanopore Technologies) with default settings, including a qscore filtering of 7. PoreChop (75) was used to remove adapters. Read quality was assessed using NanoComp (76, 77). Sequencing quality metrics are reported in Table S3 in the supplemental material.

Taxonomic classification of the filtered Nanopore reads was performed using the online BugSeq pipeline (78) and “NCBI nt” index (run 24 March 2021), which runs with a minimum read length of 100 bp and a default low-complexity filter.

### Quasimetagenomic shotgun sequencing on Illumina MiSeq.

For selected samples from the L. monocytogenes culture enrichment experiment with background microbiota harvested after 4 h of enrichment in Half Fraser, 200 ng of genomic DNA treated with MDA was subjected to paired-end sequencing (2 × 300 bp). Briefly, libraries were prepared as described in the Illumina DNA prep reference guide (Illumina DNA prep kit; Nextera DNA CD indexes; Illumina). Samples were purified, quantified with Qubit HS dsDNA (Invitrogen), normalized, and pooled. The sample pool was purified, quantified, and diluted to 4 nM prior to a denaturation and dilution procedure provided by Illumina. Illumina reads were filtered on q15 and trimmed of adaptors using fastq-mcf from the ea-utils package (79).

Taxonomic classification of the filtered Illumina reads was performed using the *k*-mer approach employed in Kraken2 v2.1.1 (80) and the available pre-built Kraken2 database PlusPFP (containing indexes for the archaea, bacteria, viral, plasmid, protozoa, fungi, plant, human, and UniVec_Coreplus RefSeq databases from 27 January 2021). A confidence score threshold of 0.05 was selected, and the minimum base quality used in classification was 20. Subsequent estimation of species abundance was performed using Bracken (81) with a threshold set to ignore species with fewer than 10 classified reads. KrakenTools (82) was used to exclude reads classified to Taxon ID 9606 (Homo sapiens) prior to submission of Illumina reads to the Sequence Read Archive (SRA).

### Mapping of *Listeria* reads to MLST database.

Illumina reads classified to Taxon IDs 1637 (*Listeria* spp.) and 1639 and below (L. monocytogenes classified to species and strain level) using Kraken2 were extracted to file using the KrakenTools extract_kraken_reads.py script (83).

The KMA mapping method (35), built to efficiently map raw sequencing data to redundant databases such as those comprised of MLST alleles, was used to map reads to the Institute Pasteur’s L. monocytogenes MLST database (59) (https://bigsdb.pasteur.fr/listeria/listeria.html). The identification of the MLST alleles using KMA was performed with the method publicly available at https://cge.cbs.dtu.dk/services/MLST/ (84) but with the program installed on our own server (downloaded from https://bitbucket.org/genomicepidemiology/mlst/src/master/).

### Data availability.

The shotgun sequencing data have been deposited in the National Center for Biotechnology Information (NCBI) archives under BioProject accession no. PRJNA721942.
