# Supplementary material for: Surveillance of Listeria monocytogenes: Early Detection, Population Dynamics, and Quasimetagenomic Sequencing during Selective Enrichment
Source: Appl Environ Microbiol. 2021 Nov 24;87(24):e01774-21. doi: 10.1128/AEM.01774-21 (PMC8612253; doi:10.1128/AEM.01774-21)
Supplement: Supplemental file 1 — Fig. S1 to S5, Tables S1 to S5. Download aem.01774-21-s0001.pdf, PDF file, 0.9 MB [file aem.01774-21-s0001.pdf]

Supplemental Material

Surveillance of *Listeria monocytogenes*: Early detection, population dynamics and quasimetagenomic sequencing during selective enrichment

Eva Wagner, Annette Fagerlund, Solveig Langsrud, Trond Møretrø, Merete Rusås Jensen, Birgitte Moen

Contents

Supplemental Figures 2

Fig. S1. Development of the microbiota in the enrichment cultures containing seven *L. monocytogenes* strains. 2

Fig. S2. Growth curves of individual *L. monocytogenes* strains. 3

Fig. S3. The effect of MDA on the relative abundance of each ST determined by *dapE* amplicon sequencing. 4

Fig. S4. The frequency of each *L. monocytogenes* ST determined by *dapE* amplicon sequencing... 5

Fig. S5. The frequency of each species determined by 16S rRNA amplicon sequencing. 6

Supplemental Tables 7

Table S1. Qualitative qPCR results obtained during enrichment of *L. monocytogenes* in the presence of background microbiota. 7

Table S2. Comparison of *L. monocytogenes* concentrations obtained by plate count and quantitative application of qPCR during enrichment in the presence of background microbiota. 9

Table S3. Summary of quality metrics for the Nanopore sequencing data. 10

Table S4. MLST profiles for the seven *L. monocytogenes* strains........................ 11

Table S5. Mapping of reads to MLST database using KMA. 12

Supplemental Figures

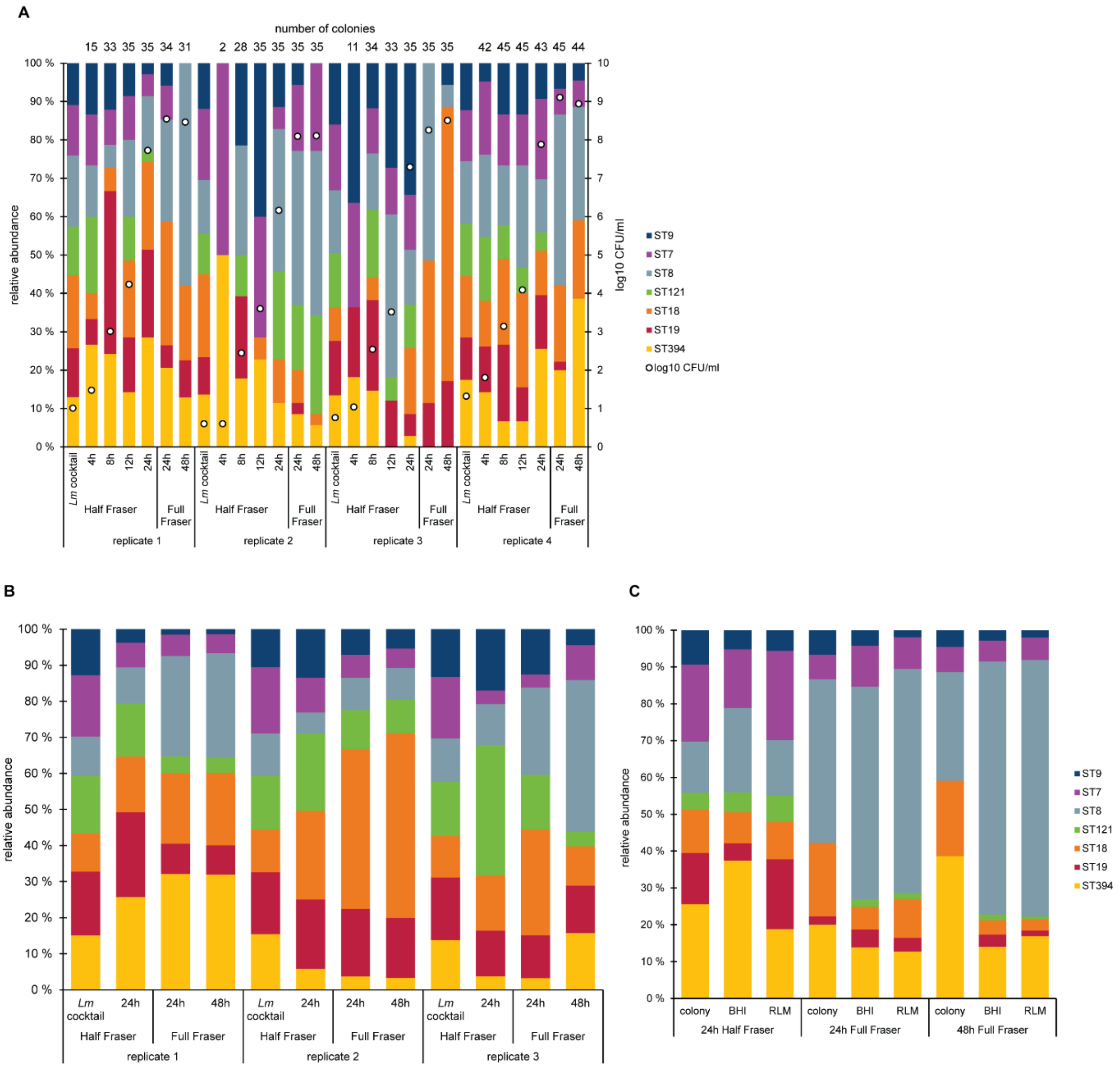

Fig. S1. Development of the microbiota in the enrichment cultures containing seven *L. monocytogenes* strains.

The proportion of each *L. monocytogenes* ST was determined by sequencing the *dapE* gene either using **(A)** PCR and subsequent Sanger sequencing of individual colonies or **(B)** amplicon sequencing of DNA isolated from culture pellets or **(C)** PCR and subsequent Sanger sequencing of individual colonies and *dapE* amplicon sequencing of all colonies scraped off a non-selective (brain heart infusion; BHI) and a *Listeria*-selective (RAPID' *L.mono*; RLM) agar plate. Individual results from **(A)** four or **(B)** three or **(C)** one (replicate 4 of the experiment) independent experiment(s) are presented.

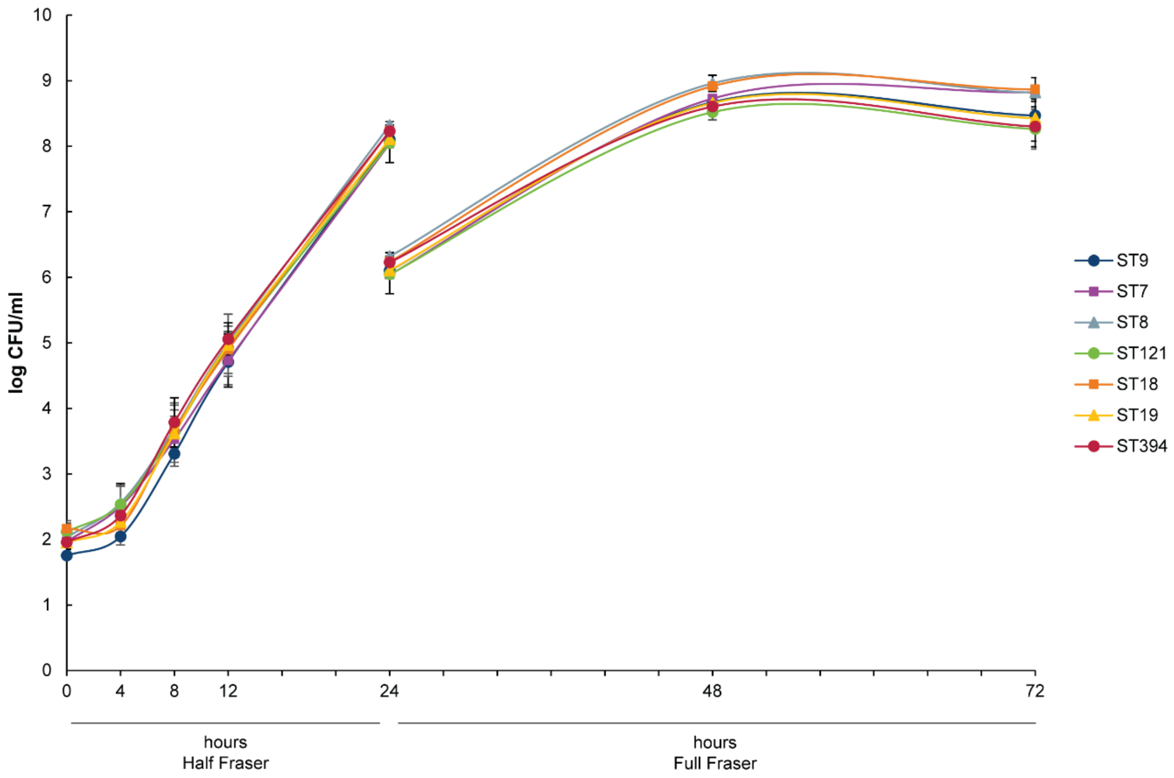

Fig. S2. Growth curves of individual *L. monocytogenes* strains.
Strains were grown in Half Fraser (30 °C for 24 h) and diluted 1:100 and further grown in Full Fraser (37 °C for 48 h). Growth curves were established by plating. Average values for three experiments are shown. Error bars denote the standard deviation.

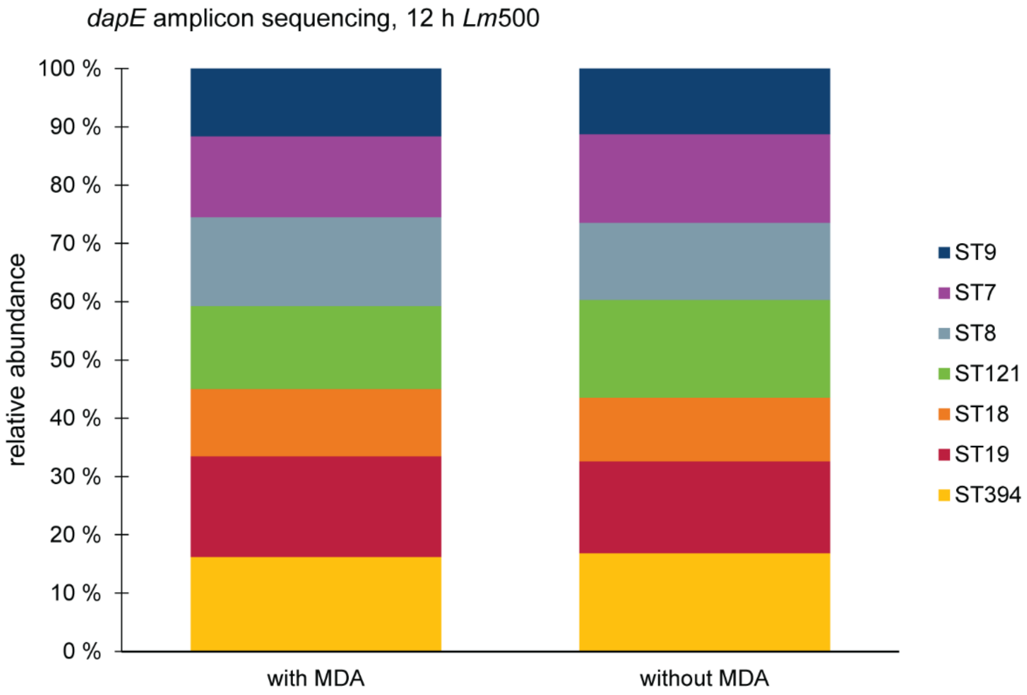

Fig. S3. The effect of MDA on the relative abundance of each ST determined by *dapE* amplicon sequencing.

dapE amplicon sequencing was performed with and without MDA on the 12 h samples from the *Lm*500 cultures from replicate 1 and replicate 3 of the enrichment experiment with background microbiota. Average relative abundances of these two experiments are shown.

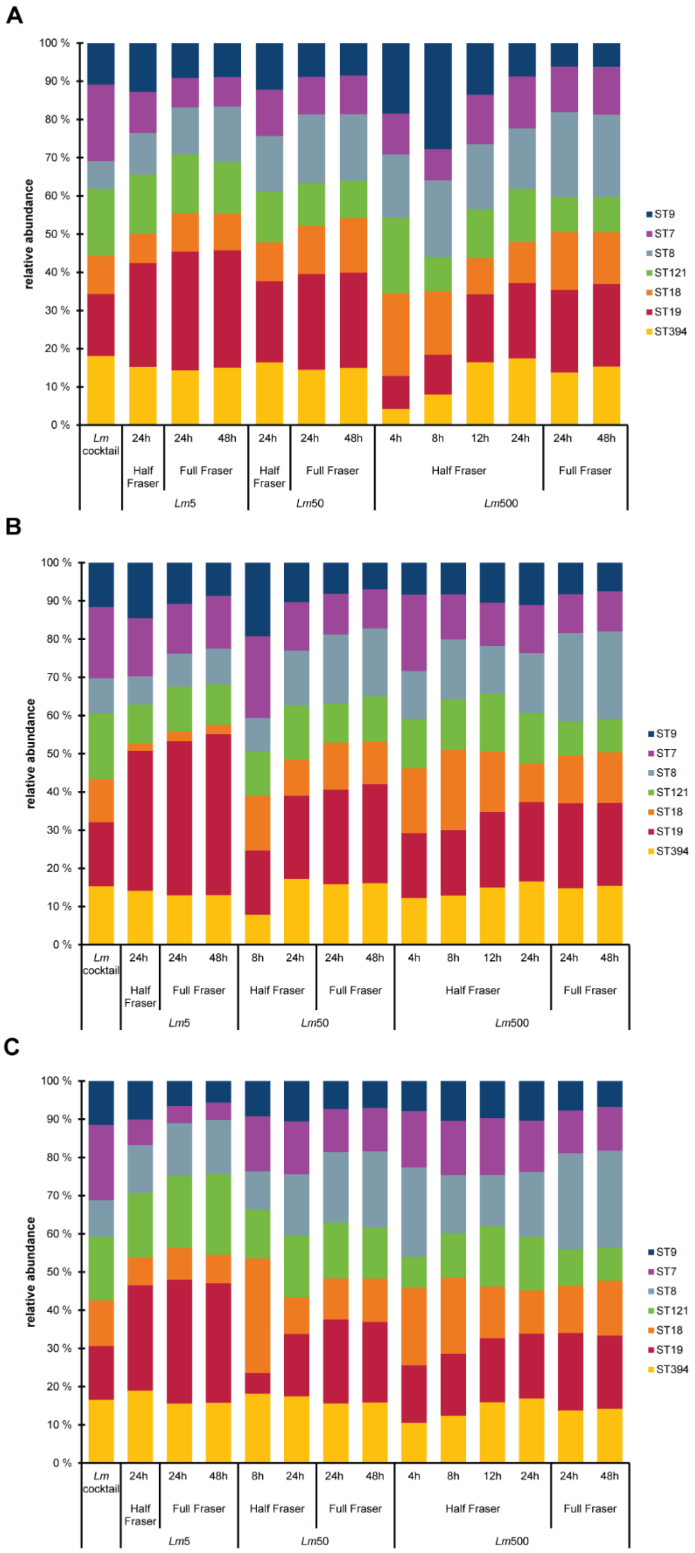

Fig. S4. The frequency of each *L. monocytogenes* ST determined by *dapE* amplicon sequencing.

The *Lm* cocktail sample constituted a mixture of all seven *L. monocytogenes* cultures in equal proportions. Presented results were obtained from the individual replicates of the experiment: **(A)** replicate 1, **(B)** replicate 2 and **(C)** replicate 3.

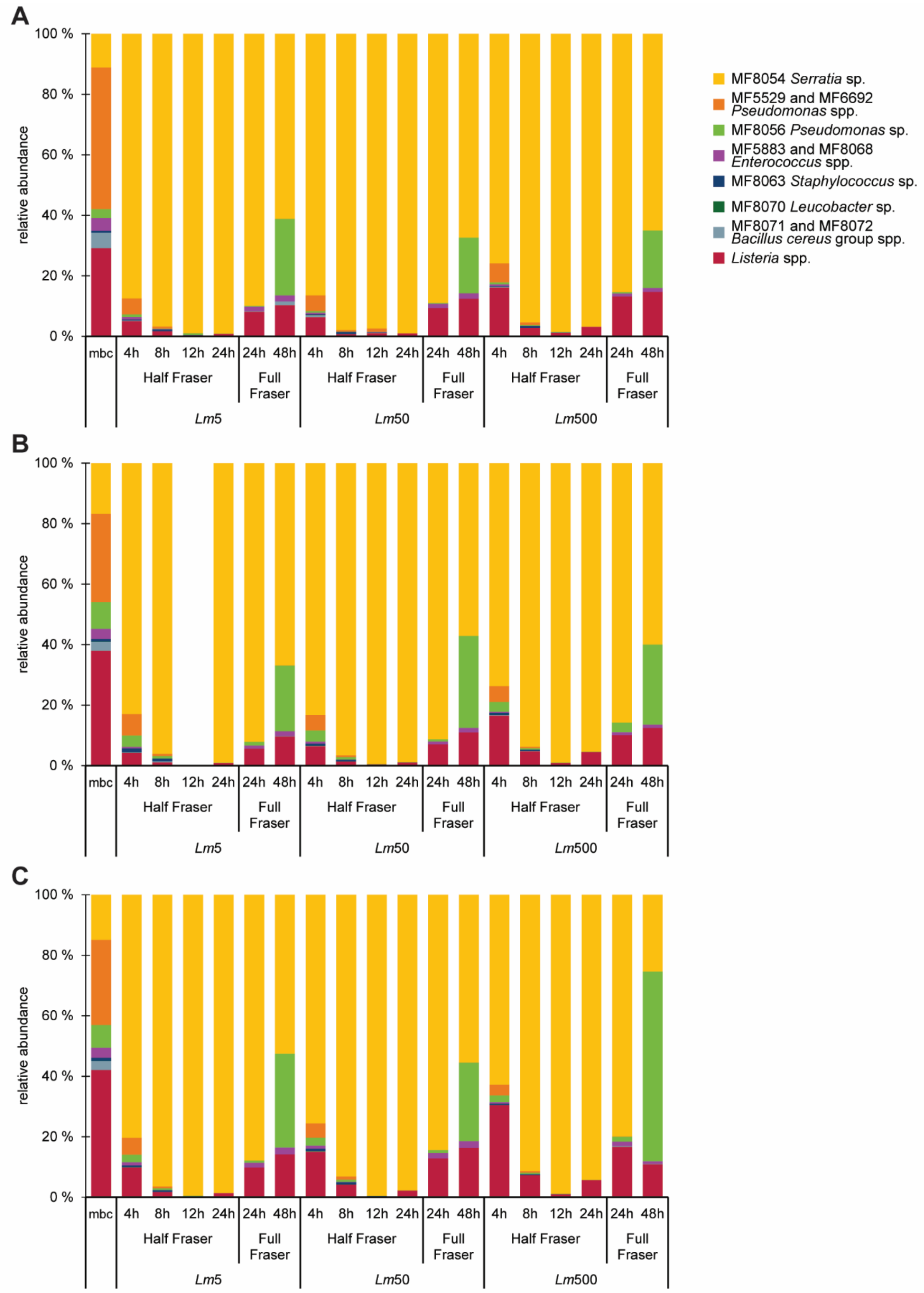

Fig. S5. The frequency of each species determined by 16S rRNA amplicon sequencing.

The microbiota cocktail sample (mbc) constituted a mixture of all *L. monocytogenes* and background microbiota cultures in equal proportions. Presented results were obtained from the individual replicates of the experiment: **(A)** replicate 1, **(B)** replicate 2 and **(C)** replicate 3. The concentration of the PCR product of the *Lm*5 12 h sample of replicate 2 was too low for sequencing.

Supplemental Tables

Table S1. Qualitative qPCR results obtained during enrichment of *L. monocytogenes* in the presence of background microbiota.

			L. monocytogenes	***Listeria* spp.**
replicate 1	4h Half Fraser	*Lm*5	negative	negative
		*Lm*50	negative	negative
		*Lm*500	negative	negative
replicate 1	8h Half Fraser	*Lm*5	negative	negative
replicate 2		*Lm*5	negative	negative
replicate 3		*Lm*5	negative	negative
replicate 1		*Lm*50	negative	negative
replicate 2		*Lm*50	negative	negative
replicate 3		*Lm*50	negative	negative
replicate 1		*Lm*500	positive	negative
replicate 2		*Lm*500	positive	positive
replicate 3		*Lm*500	positive	positive
replicate 1	12h Half Fraser	*Lm*5	negative	positive
replicate 2		*Lm*5	negative	positive
replicate 3		*Lm*5	positive	positive
replicate 1		*Lm*50	positive	positive
replicate 2		*Lm*50	positive	positive
replicate 3		*Lm*50	positive	positive
replicate 1		*Lm*500	positive	positive
replicate 2		*Lm*500	positive	positive
replicate 3		*Lm*500	positive	positive
replicate 1	24h Half Fraser	*Lm*5	positive	positive
replicate 2		*Lm*5	positive	positive
replicate 3		*Lm*5	positive	positive
replicate 1		*Lm*50	positive	positive
replicate 2		*Lm*50	positive	positive
replicate 3		*Lm*50	positive	positive
replicate 1		*Lm*500	positive	positive
replicate 2		*Lm*500	positive	positive
replicate 3		*Lm*500	positive	positive
replicate 1	24h Full Fraser	*Lm*5	positive	positive
replicate 2		*Lm*5	positive	positive
replicate 3		*Lm*5	positive	positive
replicate 1		*Lm*50	positive	positive
replicate 2		*Lm*50	positive	positive
replicate 3		*Lm*50	positive	positive
replicate 1		*Lm*500	positive	positive

replicate 2		*Lm*500	positive	positive
replicate 3		*Lm*500	positive	positive
replicate 1	48h Full Fraser	*Lm*5	positive	positive
replicate 2		*Lm*5	positive	positive
replicate 3		*Lm*5	positive	positive
replicate 1		*Lm*50	positive	positive
replicate 2		*Lm*50	positive	positive
replicate 3		*Lm*50	positive	positive
replicate 1		*Lm*500	positive	positive
replicate 2		*Lm*500	positive	positive
replicate 3		*Lm*500	positive	positive
replicate 1		negative control	negative	negative
replicate 3		negative control	negative	negative

Table S2. Comparison of *L. monocytogenes* concentrations obtained by plate count and quantitative application of qPCR during enrichment in the presence of background microbiota.

L. *monocytogenes* concentrations were obtained by plate count and qPCR during enrichment with background microbiota in Half Fraser and Full Fraser. The *L*. *monocytogenes* concentrations obtained by qPCR were calculated using standard curves established for each of the replicates. DNA was isolated from a cocktail composed of equal proportions of cultures of all *L*. *monocytogenes* strains and quantified for every replicate. The DNA concentrations were correlated with the *L*. *monocytogenes* concentrations of the same cocktails obtained by plate count. Standards were prepared by serial 1:10 from log7 CFU/reaction to 1 CFU/reaction. The obtained C_T values for the standards were then used to establish the standard curves with the known log CFU/µl in the reactions and the resulting equations were used to calculate the log CFU/µl from the C_T values of the samples.

			L. monocytogenes **log CFU/µl**	
			**plate count**	**qPCR**
replicate 1	24h Half Fraser	*Lm*5	4.68	3.83
replicate 2			4.79	3.66
replicate 3			4.74	4.70
replicate 1		*Lm*50	5.07	4.01
replicate 2			5.01	3.83
replicate 3			4.95	4.99
replicate 1		*Lm*500	5.26	4.50
replicate 2			5.34	4.35
replicate 3			5.47	5.32
replicate 1	24h Full Fraser	*Lm*5	5.58	4.94
replicate 2			5.54	4.76
replicate 3			5.45	5.65
replicate 1		*Lm*50	5.61	4.99
replicate 2			5.52	4.76
replicate 3			5.34	5.68
replicate 1		*Lm*500	5.87	5.05
replicate 2			5.82	4.91
replicate 3			5.74	5.88
replicate 1	48h Full Fraser	*Lm*5	4.95	4.73
replicate 2			4.96	4.70
replicate 3			5.29	5.67
replicate 1		*Lm*50	5.32	4.82
replicate 2			5.11	4.77
replicate 3			5.43	5.75
replicate 1		*Lm*500	5.48	4.93
replicate 2			5.38	4.72
replicate 3			5.42	5.84

Table S3. Summary of quality metrics for the Nanopore sequencing data. Data obtained after filtering on Q≥7 and running PoreChop to remove adapters are presented.

Sample			Length of sequencing run (hours)	All reads			reads > 1000 bases	
Culture	Time	MDA		no. of reads	megabases	read length N50	no. of reads	megabases
***Lm*5**	4 h	+	1 h	725	0.8	1 926	270	0.6
***Lm*50**	4 h	+	5 h	7 245	7.2	1 720	2 229	5.1
***Lm*500**	4 h	+	7 h	35 209	49.5	2 671	14 049	39.9
***Lm*5**	8 h	+	4 h	15 938	26.1	3 074	7 510	22.2
***Lm*50**	8 h	+	6 h	8 705	17.2	4 268	4 175	15.2
***Lm*500**	8 h	+	4 h	5 213	9.6	4 086	2 370	8.4
***Lm*5**	12 h	+	7 h	9 918	14.1	3 063	3 839	11.6
***Lm*50**	12 h	+	2 h	2 809	5.5	4 118	1 363	4.9
***Lm*500**	12 h	+	4 h	5 799	10.0	3 900	2 450	8.6
***Lm*500**	12 h	-	15 h	3 246	7.2	3 909	1 960	6.6
***Lm*500**	24 h	-	9 h	47 331	92.5	5 232	19 703	81.9

Table S4. MLST profiles for the seven *L. monocytogenes* strains.
Allele numbers and profiles for each strain are reported.

	**ST**	***abcZ***	***bglA***	***cat***	***dapE***	***dat***	***ldh***	***lhkA***
MF5376	**ST7**	5	8	5	7	6	2	1
MF5377	**ST8**	5	6	2	9	5	3	1
MF4536	**ST9**	6	5	6	4	1	4	1
MF4565	**ST18**	7	6	15	18	12	6	1
MF5630	**ST19**	7	6	19	6	1	24	1
MF5634	**ST121**	7	6	8	8	6	37	1
MF5378	**ST394**	5	5	17	21	39	2	6
combined		5, 6, 7	5, 6, 8	2, 5, 6, 8, 15, 17, 19	4, 6, 7, 8, 9, 18, 21	1, 5, 6, 12, 39	2, 3, 4, 6, 24, 37	1, 6

Table S5. Mapping of reads to MLST database using KMA.

Output from mapping of Illumina reads classified as *Listeria* spp. or *L. monocytogenes*. Results for alleles expected to be present in the cultures containing the seven different *L. monocytogenes* STs (see Table S3) are indicated in bold typeface.

#Template	correct?	Score	Expected	Template length	Template Identity	Template Coverage	Query Identity	Query Coverage	Depth	q value	p value	Comment
**abcZ_5**	**1**	**7578**	**103**	**537**	**100**	**100**	**100**	**100**	**14.37**	**7274.12**	**1.00E-26**	
**abcZ_6**	**1**	**602**	**105**	**537**	**73.18**	**73.18**	**100**	**136.64**	**1.12**	**348.51**	**1.00E-26**	
**abcZ_7**	**1**	**1397**	**105**	**537**	**62.94**	**62.94**	**100**	**158.88**	**2.56**	**1110.89**	**1.00E-26**	
abcZ_168	0	217	105	537	43.2	44.32	97.48	225.63	0.44	38.48	1.10E-09	
abcZ_186	0	251	105	537	30.17	30.17	100	331.48	0.47	59.31	1.00E-13	
abcZ_441	0	238	105	537	52.33	56.05	93.36	178.41	0.56	51.04	1.00E-12	
abcZ_461	0	123	105	537	24.77	25.7	96.38	389.13	0.26	1.32	2.50E-01	
abcZ_476	0	236	105	538	22.3	22.3	100	448.33	0.44	49.62	1.00E-11	
**bglA_5**	**1**	**5322**	**77**	**399**	**100**	**100**	**100**	**100**	**13.62**	**5094.86**	**1.00E-26**	
**bglA_6**	**1**	**13989**	**75**	**399**	**100**	**100**	**100**	**100**	**35.13**	**13765.46**	**1.00E-26**	
**bglA_8**	**1**	**4002**	**77**	**399**	**100**	**100**	**100**	**100**	**10.27**	**3775.36**	**1.00E-26**	
bglA_10	0	2490	77	399	75.19	75.19	100	133	6.25	2265.8	1.00E-26	
bglA_210	0	80	78	399	23.06	24.56	93.88	407.14	0.25	0.01	9.00E-01	
bglA_212	0	339	78	399	34.59	36.09	95.83	277.08	1.04	162.68	1.00E-26	
bglA_299	0	234	78	399	62.41	63.66	98.03	157.09	0.64	77.45	1.00E-17	
bglA_311	0	204	78	399	52.63	53.38	98.59	187.32	0.53	55.81	1.00E-13	
bglA_316	0	209	78	399	53.38	53.88	99.07	185.58	0.54	59.3	1.00E-13	
bglA_428	0	156	78	399	44.11	46.62	94.62	214.52	0.47	25.64	4.10E-07	
**cat_5**	**1**	**2065**	**94**	**486**	**100**	**100**	**100**	**100**	**4.38**	**1796.72**	**1.00E-26**	
**cat_6**	**1**	**9014**	**92**	**486**	**100**	**100**	**100**	**100**	**18.46**	**8739.06**	**1.00E-26**	
**cat_8**	**1**	**2486**	**94**	**486**	**78.81**	**80.45**	**97.95**	**124.3**	**5.17**	**2215.33**	**1.00E-26**	
cat_10	0	2607	94	486	47.74	47.74	100	209.48	5.42	2335.81	1.00E-26	
**cat_17**	**1**	**592**	**95**	**486**	**47.33**	**47.33**	**100**	**211.3**	**1.22**	**358.68**	**1.00E-26**	
**cat_19**	**1**	**178**	**95**	**486**	**33.13**	**36.42**	**90.96**	**274.58**	**0.44**	**24.84**	**6.20E-07**	
cat_59	0	160	95	486	38.89	40.53	95.94	246.7	0.41	16.24	5.60E-05	
cat_122	0	438	95	486	67.49	72.84	92.66	137.29	1.03	219.9	1.00E-26	
cat_131	0	209	95	486	49.38	51.23	96.39	195.18	0.51	42.26	5.90E-10	
cat_316	0	300	95	486	61.73	61.73	100	162	0.62	105.7	1.00E-24	
cat_328	0	328	95	486	38.07	40.33	94.39	247.96	0.81	127.61	1.00E-26	
cat_420	0	127	95	486	21.4	23.25	92.04	430.09	0.32	4.43	3.50E-02	
**dapE_4**	**1**	**6989**	**88**	**462**	**90.91**	**90.91**	**100**	**110**	**15.21**	**6726.77**	**1.00E-26**	
dapE_5	0	18200	85	462	83.12	83.12	100	120.31	39.66	17944.51	1.00E-26	
**dapE_6**	**1**	**46688**	**77**	**462**	**100**	**100**	**100**	**100**	**101.68**	**46455.83**	**1.00E-26**	
**dapE_7**	**1**	**1158**	**90**	**462**	**91.34**	**91.34**	**100**	**109.48**	**2.51**	**912.58**	**1.00E-26**	
**dapE_8**	**1**	**6329**	**89**	**462**	**100**	**100**	**100**	**100**	**13.73**	**6066.68**	**1.00E-26**	
**dapE_9**	**1**	**11983**	**87**	**462**	**100**	**100**	**100**	**100**	**25.84**	**11723.12**	**1.00E-26**	
**dapE_18**	**1**	**60017**	**73**	**462**	**100**	**100**	**100**	**100**	**131.1**	**59796.1**	**1.00E-26**	
dapE_19	0	338	90	462	40.69	42.86	94.95	233.33	0.86	142.51	1.00E-26	
dapE_20	0	2880	90	462	85.5	85.5	100	116.96	6.28	2620.71	1.00E-26	
dapE_29	0	15986	86	462	79.44	79.44	100	125.89	34.83	15728.86	1.00E-26	
dapE_33	0	1020	90	462	98.05	98.05	100	101.99	2.26	777.75	1.00E-26	
dapE_78	0	196	90	462	45.45	46.97	96.77	212.9	0.47	38.55	1.00E-09	
dapE_105	0	429	90	462	22.51	23.81	94.55	420	1.04	220.09	1.00E-26	
dapE_125	0	263	90	461	61.82	64.86	95.32	154.18	0.65	84.03	1.00E-19	
dapE_127	0	114	90	462	12.77	12.99	98.33	770	0.26	2.61	1.10E-01	
dapE_143	0	273	90	462	62.99	64.94	97	154	0.65	91.23	1.00E-20	
dapE_156	0	114	90	462	12.77	12.99	98.33	770	0.26	2.61	1.10E-01	
dapE_162	0	568	90	461	79.83	80.48	99.19	124.26	1.3	346.18	1.00E-26	
dapE_165	0	286	90	462	64.07	65.15	98.34	153.49	0.65	101.1	1.00E-23	
dapE_173	0	233	90	462	59.96	65.15	92.03	153.49	0.65	62.42	1.00E-14	
dapE_180	0	193	90	462	42.64	43.07	98.99	232.16	0.43	36.77	1.80E-09	
dapE_185	0	480	90	462	51.95	51.95	100	192.5	1.04	265.45	1.00E-26	
dapE_264	0	686	90	462	63.2	69.05	91.54	144.83	1.81	456.27	1.00E-26	
dapE_276	0	1100	90	462	95.24	95.89	99.32	104.29	2.45	855.82	1.00E-26	
dapE_277	0	196	90	462	42.86	43.07	99.5	232.16	0.43	38.55	1.00E-09	
dapE_283	0	232	90	462	51.95	52.81	98.36	189.34	0.53	61.73	1.00E-14	
dapE_292	0	150	90	462	33.77	34.42	98.11	290.57	0.34	14.52	1.40E-04	
dapE_302	0	406	90	462	48.27	51.52	93.7	194.12	1.03	200.01	1.00E-26	
dapE_309	0	255	90	461	62.04	65.08	95.33	153.67	0.65	78.17	1.00E-18	
dapE_312	0	284	90	462	63.64	64.72	98.33	154.52	0.65	99.57	1.00E-22	
dapE_315	0	160	90	462	20.35	21	96.91	476.29	0.42	19.06	1.30E-05	
dapE_367	0	342	90	462	21.65	21.65	100	462	0.75	145.8	1.00E-26	
dapE_428	0	281	90	462	63.42	64.72	97.99	154.52	0.65	97.28	1.00E-22	
dapE_431	0	603	90	462	52.38	52.81	99.18	189.34	1.36	378.29	1.00E-26	
dapE_442	0	271	90	462	61.69	63.2	97.6	158.22	0.63	89.73	1.00E-20	
dapE_461	0	268	90	462	62.77	65.15	96.35	153.49	0.65	87.49	1.00E-20	
dapE_467	0	458	90	462	57.14	58.23	98.14	171.75	1.06	245.75	1.00E-26	
dapE_493	0	200	90	462	24.46	25.54	95.76	391.53	0.51	40.97	6.70E-10	
dapE_500	0	248	90	462	54.55	54.98	99.21	181.89	0.55	72.91	1.00E-16	
dapE_505	0	234	90	462	25.32	25.32	100	394.87	0.51	63.1	1.00E-14	
dapE_524	0	223	90	462	27.27	27.27	100	366.67	0.48	55.66	1.00E-13	
dapE_540	0	442	90	462	84.63	86.58	97.75	115.5	1.14	231.55	1.00E-26	
dapE_564	0	322	90	462	42.21	45.67	92.42	218.96	0.91	129.48	1.00E-26	
**dat_1**	**1**	**2067**	**92**	**471**	**92.78**	**92.78**	**100**	**107.78**	**4.33**	**1806.51**	**1.00E-26**	
**dat_12**	**1**	**600**	**92**	**471**	**79.83**	**79.83**	**100**	**125.27**	**1.27**	**371.94**	**1.00E-26**	
**dat_39**	**1**	**260**	**92**	**471**	**27.6**	**27.6**	**100**	**362.31**	**0.55**	**79.48**	**1.00E-18**	
**ldh_2**	**1**	**2844**	**88**	**453**	**78.37**	**78.37**	**100**	**127.61**	**6.42**	**2589.66**	**1.00E-26**	
**ldh_3**	**1**	**30991**	**80**	**453**	**100**	**100**	**100**	**100**	**69.61**	**30750.5**	**1.00E-26**	
**ldh_6**	**1**	**5001**	**87**	**453**	**91.39**	**93.38**	**97.87**	**107.09**	**11.25**	**4743.88**	**1.00E-26**	
ldh_7	0	4445	87	453	95.36	95.36	100	104.86	9.98	4188.17	1.00E-26	
**ldh_24**	**1**	**1611**	**88**	**453**	**67.77**	**67.77**	**100**	**147.56**	**3.56**	**1363.48**	**1.00E-26**	
**ldh_37**	**1**	**1089**	**88**	**453**	**57.62**	**57.62**	**100**	**173.56**	**2.41**	**849.33**	**1.00E-26**	
ldh_40	0	786	88	453	54.53	55.85	97.63	179.05	1.84	555.42	1.00E-26	
ldh_141	0	182	89	453	43.27	44.81	96.55	223.15	0.45	31.86	1.70E-08	
ldh_158	0	183	89	453	43.49	45.03	96.57	222.06	0.45	32.43	1.30E-08	
ldh_234	0	570	88	453	64.46	64.68	99.66	154.61	1.29	351.15	1.00E-26	
ldh_240	0	12798	85	453	99.78	100	99.78	100	29.02	12543.65	1.00E-26	*L. innocua*
ldh_249	0	817	88	453	56.07	56.07	100	178.35	1.87	585.21	1.00E-26	
ldh_263	0	288	89	453	65.78	66.23	99.33	151	0.66	104.98	1.00E-23	

ldh_268	0	367	89	453	47.68	50.77	93.91	196.96	1.02	169.45	1.00E-26
ldh_279	0	256	89	453	61.81	64.46	95.89	155.14	0.64	80.77	1.00E-18
ldh_416	0	182	89	453	42.38	43.49	97.46	229.95	0.43	31.86	1.70E-08
ldh_438	0	275	89	453	60.71	60.71	100	164.73	0.61	94.98	1.00E-21
ldh_461	0	248	89	453	58.72	60.71	96.73	164.73	0.61	74.95	1.00E-17
ldh_549	0	120	89	453	30.46	32.45	93.88	308.16	0.32	4.57	3.30E-02
ldh_642	0	243	89	453	61.81	66.23	93.33	151	0.66	71.37	1.00E-16
**lhkA_1**	**1**	**10173**	**91**	**480**	**100**	**100**	**100**	**100**	**21.44**	**9902**	**1.00E-26**
**lhkA_6**	**1**	**947**	**94**	**480**	**57.71**	**57.71**	**100**	**173.29**	**1.97**	**698.59**	**1.00E-26**
lhkA_78	0	120	94	480	28.33	30.62	92.52	326.53	0.31	3.06	8.00E-02
lhkA_208	0	163	94	480	39.17	42.5	92.16	235.29	0.42	18.29	1.90E-05
lhkA_225	0	329	94	480	39.58	43.54	90.91	229.67	0.79	130.07	1.00E-26
lhkA_274	0	154	94	480	38.54	42.5	90.69	235.29	0.42	14.3	1.60E-04
lhkA_396	0	746	94	480	91.04	91.46	99.54	109.34	1.63	505.61	1.00E-26
lhkA_417	0	657	94	480	11.46	12.5	91.67	800	1.45	421.56	1.00E-26
